# Biomarker Prediction of Delayed Graft Function and Prognosis Post–Kidney Transplantation

**DOI:** 10.1016/j.ekir.2025.10.021

**Published:** 2025-11-05

**Authors:** Rosamonde E. Banks, Michelle Wilson, Matthew Welberry Smith, Andrew J.P. Lewington, Mary Jo Kurth, Helen Sewell, Rebecca Bartle, Joanne M. Watt, Mark W. Ruddock, Damien McAleer, Hanagh Winter, Peter Fitzgerald, Paul Gibbs, Neil S. Sheerin, Colin Jones, John Stoves, Dan Ridgway, William S. McKane, Anusha Edwards, Sunil Bhandari, Matthew Edey, Douglas Thompson, Carys M. Lippiatt, Peter J. Selby

**Affiliations:** 1 Leeds Institute for Medical Research at St James’s, University of Leeds, Leeds, UK; 2 Department of Renal Medicine, St. James’s University Hospital, Leeds, UK; 3 Clinical Studies Group, Randox Laboratories Ltd., Crumlin, UK; 4 Randox Laboratories Ltd., Crumlin, UK; 5 Portsmouth Hospitals NHS Trust, Portsmouth, UK; 6 Newcastle Biomedical Research Centre and the NIHR Blood and Transplant Research Unit in Organ Donation and Transplantation, Newcastle University, Newcastle, UK; 7 York and Scarborough Teaching Hospitals NHS Foundation Trust, York, UK; 8 Bradford Renal Unit, Bradford Teaching Hospitals, NHS Foundation Trust, Bradford, UK; 9 Department of Transplant Surgery, Liverpool University Hospitals NHS Foundation Trust, Liverpool, UK; 10 Sheffield Teaching Hospitals NHS Foundation Trust, Sheffield, UK; 11 North Bristol NHS Trust, Southmead Hospital, Bristol, UK; 12 Hull University Teaching Hospitals NHS Trust and Hull York Medical School, Hull, UK; 13 Department of Blood Sciences, St. James’s University Hospital, Beckett Street, Leeds, UK; 14 Department of Specialist Laboratory Medicine, St. James’s University Hospital, Beckett Street, Leeds, UK

**Keywords:** aminoacylase-1, cystatin C, delayed graft function, kidney transplantation, midkine, sTNFR1

## Abstract

**Introduction:**

Aminoacylase-1 (ACY1) and additional biomarkers were evaluated for prediction of delayed graft function (DGF) and prognosis following kidney transplantation.

**Methods:**

Serum biomarkers were measured (days 1–2 posttransplant for DGF prediction and 1–3 for prognosis) in 237 patients transplanted in Leeds (2003–2011) in the discovery phase, and 319 patients from 7 UK transplant centers (2012–2016) in the validation phase. Median follow-up was 13.28 years (interquartile range [IQR]: 12.4–13.8) and 9.03 years (IQR: 5.19–10.16), respectively.

**Results:**

DGF occurred in 29.5% of discovery cohort patients and 18.2% of validation cohort patients. A DGF linear predictor combining ACY1, soluble tumor necrosis factor receptor-1 (sTNFR1) and cystatin C (CysC) demonstrated an area under the receiver operating characteristic (AUROC) of 0.93, decreasing to 0.83 during validation. Comparable values for the individual components were ACY1 (0.79 vs. 0.65), sTNFR1 (0.88 vs. 0.89), CysC (0.89 vs. 0.82), and 0.75 vs. 0.81 for serum creatinine (Cr) as the gold standard. The linear predictor variables for death-censored graft survival (DCGS) were CysC, ACY1, midkine, and recipient age at transplant, but k-statistic values of 0.55 in all transplants and 0.52 in deceased donor kidney transplants (DDKT) precluded validation. Individually, sTNFR1, CysC, and Cr were significantly associated with DCGS in both the discovery and validation phases. Preliminary findings indicated a consistent association of ACY1 and CysC with DCGS in DDKTs affected by DGF. Impacts of clinical practice changes and biomarker performance across the 2 phases are described.

**Conclusion:**

Several biomarkers show potential as predictors of DGF or outcome and should be explored further.

## Graphical abstract

**Figure:**
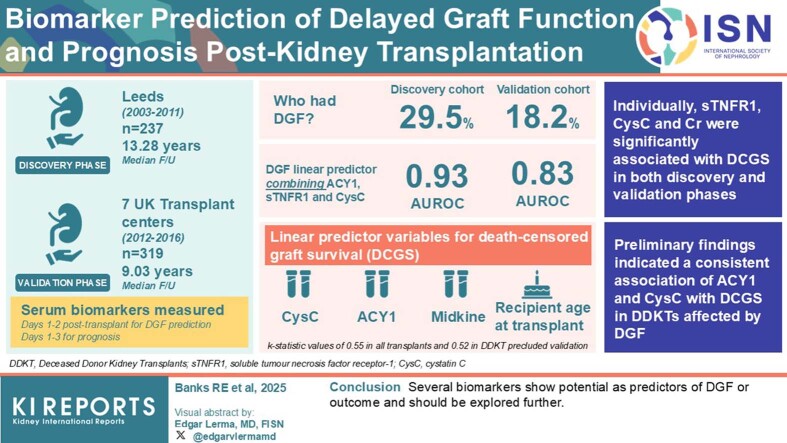

Globally, 118,322 kidney transplants were performed in 2023.^1^ The increasing complexity of recipient demographics and the use of kidneys with increased risk of complications such as DGF, has major financial implications.^2^ Defined most frequently as dialysis in the first week posttransplant,^3^^,^^4^ DGF is predominantly attributed to ischemia-reperfusion injury.^4^^,^^5^ Primarily affecting DDKTs, DGF incidence ranges from 19% to 91%, reflecting variability in definitions, donor type, and the subjective nature of dialysis decisions.^3^, ^4^, ^6^ Factors most strongly associated with DGF include donation after circulatory death (DCD), increased cold ischemia time (CIT), and donor Cr and age.^6^^,^^7^ Consequences include acute rejection, increased morbidity, prolonged hospital stay, and impacts on transplant function and loss, although these are influenced by donor type and DGF duration,^6^^,^^8^, ^9^, ^10^, ^11^, ^12^, ^13^, ^14^, ^15^, ^16^, ^17^ with a recent proposal to refine DGF based on dialysis duration.^6^

The clinical decision to initiate dialysis posttransplant is largely subjective, based on serial Cr, fluid balance assessments, and potassium levels. Biomarkers allowing objective prediction of DGF could aid patient management, provide patient outcome and economic benefits, and enable trial enrichment strategies in emerging clinical trials.^2^^,^^18^, ^19^, ^20^, ^21^, ^22^ Prognostic biomarkers may allow earlier intervention and improve longer-term outcomes.^23^ Despite many transplant-related biomarker studies, translation to clinical use remains challenging.^24^^,^^25^

Previously, we showed serum levels of the novel biomarker ACY1 and CysC on days 1 to 2 posttransplant were significant predictors of DGF.^26^ Importantly, in patients with DGF, serum ACY1 was prognostic for graft loss. Addressing the challenges of biomarker development,^25^ the primary aims of this study were to transfer the ACY1 assay to the Randox Laboratories biochip platform and further explore ACY1 and additional potential biomarkers individually, or in a multivariable model, in (i) prediction of DGF and (ii) long-term prognosis, using a discovery and independent validation cohort.

## Methods

### Patient Cohorts and Sample Collection

**Figure 1:**
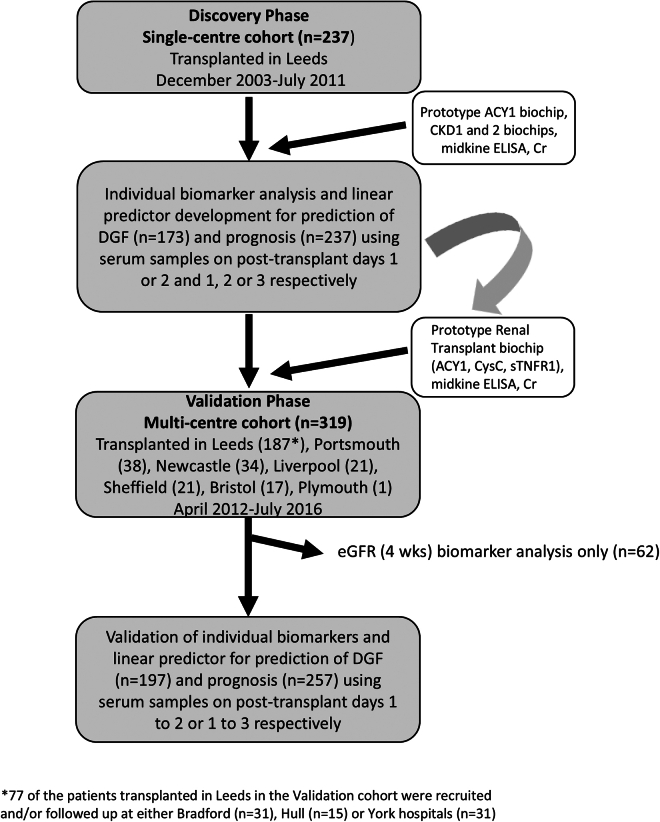
Schematic showing the patient cohorts and study design for the BioPAsSPoRT (Biomarkers for patient assessment and stratification post–renal transplantation) study. ACY1, aminoacylase-1; Cr, creatinine; CysC, cystatin C; DGF, delayed graft function; eGFR, estimated glomerular filtration rate; ELISA, enzyme-linked immunosorbent assay; sTNFR1, soluble tumor necrosis factor receptor-1.

The study design is summarized in Figure 1. For the discovery phase, serum samples (days 1–3 posttransplant) were obtained from the Leeds Multidisciplinary Research Tissue Bank (RTB) from 237 patients undergoing kidney transplantation in Leeds (2003–2011; 80% in 2003–2006).^26^ For the independent validation phase, prospectively collected samples were obtained from the Leeds NIHR BioRTB from 319 patients transplanted (2012–2016) in 7 UK transplant centers.^27^ Ethical approval was in place, and all patients gave informed consent. Samples were processed following a standardized protocol across both cohorts, stored at −80 ^o^C and comprehensive clinical data collected.^26^^,^^27^

### Discovery Phase—Biomarker Analysis for Prediction of DGF or Prognosis

Biomarkers were measured in day 1 or 2 posttransplant serum samples for DGF prediction and day 1, 2, or 3 for prognosis (*n* = 237; Supplementary Table S1) with day 0 being the day of transplant reperfusion. Measurements were performed in Leeds using the Randox Evidence Investigator (Randox Laboratories Ltd, Crumlin, UK) with samples analyzed in duplicate, blinded, and randomized. A prototype ACY1 biochip was used and additional proteins of potential relevance (Supplementary Table S2) including macrophage inflammatory protein 1-alpha, sTNFR1 and sTNFR2, and neutrophil gelatinase-associated lipocalin (NGAL) were measured using Chronic Kidney Disease Arrays 1 and 2 (Randox Laboratories Ltd). Midkine (potential biomarker for acute kidney disease) was measured at Randox Laboratories by enzyme-linked immunosorbent assay according to manufacturer’s instructions (Lyramid, Sydney, Australia). Serum Cr and CysC were measured in the Blood Sciences Department at Leeds Teaching Hospitals NHS Trust (Siemens).^26^ Biomarkers were also profiled in 15 patients pretransplant and at intervals up to day 25 posttransplant, and in 7 live donors before and after kidney donation to assess any potential surgery and nephrectomy-associated impact (visualized using GraphPad Prism V10.5.0).

### Validation Phase

A prototype renal transplant panel (RTP) biochip for measuring ACY1, sTNFR1, and CysC was manufactured and validated technically (Randox Laboratories Ltd) including assessment of limits of detection and limits of quantification (LOQ), precision, analyte specificity, linearity of dilution, interferences and assay stability. Posttransplant serum samples from 257 patients in the independent validation cohort (Figure 1, Supplementary Table S1) were analyzed on the RTP biochip at Leeds using the Randox Evidence Investigator. Serum Cr was determined at each center and midkine by enzyme-linked immunosorbent assay at Randox Laboratories Ltd. To explore any relationship between estimated glomerular filtration rate (eGFR) and biomarkers, serum samples collected at 4 weeks ± 4 days posttransplant (*n* = 276) were also analyzed.

### Statistical Analyses

Statistical analysis was performed using R^28^ Version 4.4.2 in R Studio^29^ and reported according to TRIPOD guidelines.^30^ *P*-values < 0.05 were considered significant. Nonparametric tests were used to explore differences within and between cohorts. Biomarker concentrations were assessed in terms of distributions, outliers, missing values and collinearity. Measurements above and below LOQs were replaced with upper LOQ (ULOQ) and lower LOQ (LLOQ) values, respectively.

#### Discovery Phase

##### Outcomes

The primary outcomes were the following: (i) development of DGF, defined as dialysis in the first 7 days posttransplant, excluding for isolated hyperkalaemia,^22^^,^^23^^,^^31^ and (ii) DCGS with graft loss defined as long-term return to dialysis or preemptive retransplantation. Patients not requiring dialysis or lost to follow-up were censored on the last date known to be dialysis-free, and patients who died were censored on date of death.

##### Missing Data

Missing biomarker data was imputed using multiple imputation (*n* = 20) by chained equations implemented via the “mice” function in the R package MICE^32^ assuming data were missing at random. This involved Cr and ACY1 (*n* = 1), sTNFR1 (*n* = 2), CysC (*n* = 14), midkine (*n* = 23), interleukin-8 and fatty acid-binding protein 1 (*n* = 11), and epidermal growth factor (EGF; *n* = 13). Imputation models were run for each outcome and included all potential predictor variables and respective outcome variables.

##### Univariable Associations

Associations between biomarkers or clinical factors and DGF were explored using univariable logistic regression.^33^ For comparison between non-DGF and DGF, the Wilcoxon rank-sum test or Kruskal-Wallis rank test for continuous variables and the chi-square test for categorical variables and logistic regression were used. Associations with DCGS were visualized using Kaplan-Meier plots, with continuous variables dichotomized around a cut-point derived to achieve optimal concordance and the log-rank test used to identify significant differences and Cox PH. The linearity assumption was assessed using visual inspection of Martingale residuals and, where appropriate, transformations were applied using the fractional polynomial method.^33^ The proportional hazards assumption was tested using Schoenfeld residuals.^34^

##### Predictors and Initial Shortlisting

Potential predictors considered for both outcomes included 9 biomarkers (ACY1, EGF, fatty acid binding protein 1, sTNFR1, interleukin-8, NGAL, CysC, Cr, and midkine), and 6 clinical variables selected based on previous reports and data availability (recipient age, donor type, total human leukocyte antigen mismatch, CIT, warm ischemic time, and number of previous transplants). Integral to the biochips but excluded were C-reactive protein (surgical impacts), macrophage inflammatory protein 1-alpha (inappropriate assay range), D-dimer and C3a (matrix unsuitability), and sTNFR2 (range considerations, significant correlation and overlapping profiles with sTNFR1). Continuous variables were logged before analysis. Assuming a requirement of 5 to 10 events per variable,^35^ multivariable model development containing these 15 variables (16 parameters) requires at least 80 events.

To shortlist biomarkers and clinical variables for both DGF prediction and prognosis, initial selection from the 15 was carried out using penalized LASSO regression for both outcomes (logistic for DGF and survival for DCGS) using cv.glmnet in the glmnet package.^36^ Models were run in each imputed dataset. Coefficients for tuning parameters lambda min (minimum cross-validated error [cvm]) and lambda 1 SE (largest lambda such that error is within 1 SE of the minimum cvm) were extracted and averaged across imputations, resulting in 4 sets of coefficients, that is, 2 for each outcome. Variables were then shortlisted for the final model derivations.

##### Derivation of Final Models

Variable selection was repeated for each outcome using the final shortlisted biomarkers and clinical predictors. Models were run in all 20 imputed datasets, coefficients for the tuning parameter lambda 1 SE were extracted and averaged to determine 1 final set of model coefficients for each outcome. Coefficients derived were used to calculate a linear predictor for each patient in each imputed data set and these were then averaged to obtain a single linear predictor for each outcome.

##### Prediction of DGF

AUROC was calculated in each imputation using the R package pROC*^37^* with calculation of SE using se_auc in the auctestr package.*^38^* Estimates were pooled and 95% confidence intervals obtained using the function pool_auc from the psfmi package.*^39^* An optimal cut-point was derived by maximizing the Youden Index and sensitivity, specificity and predictive values calculated. AUROCs and optimal cut-points were derived for each biomarker and compared with the multivariable linear predictor using roc.test in the pROC package.

##### Prognosis – DCGS

Gönen and Hellers K-statistic*^40^* (less biased because it is not dependent on censoring distribution)*^41^* and SE were calculated in each imputation using phcpe in the CPE package*^42^* and then pooled using the function pool_RR from the psfmi package.*^43^* Baseline graft survival at 1, 5, and 10 years were estimated in each imputation using survfit from the survival package*^44^* and averaged giving 1 estimate of each. Kaplan-Meier plots were produced to explore the predictive utility of the final model, using the averaged linear predictor, and of the individual markers using adjustedsurv in the adjustedCurves package*^45^* when stratifying by risk of graft loss. Stratification thresholds were derived for optimal K and log-rank tests performed.

Both outcomes were explored in all patients with available samples and in DDKTs only. Overfitting was assessed using bootstrapping (*n* = 200) with replacement.

### Validation Phase

The linear predictors developed in the discovery phase were applied to the independent validation cohort and discrimination and calibration assessed. Individual biomarkers selected for this phase were also assessed. Using the powerROC tool^46^ with AUROC of 0.93 and DGF frequency of 18% and default of 0.1 for 95% CI width requires 187 patients (33 events), increasing to 318 (57 events) if using a conservative estimate of a 5% decline in AUROC to 0.88.

## Results

### Discovery Phase

#### Patient Cohort and Clinical Associations

**Figure 2:**
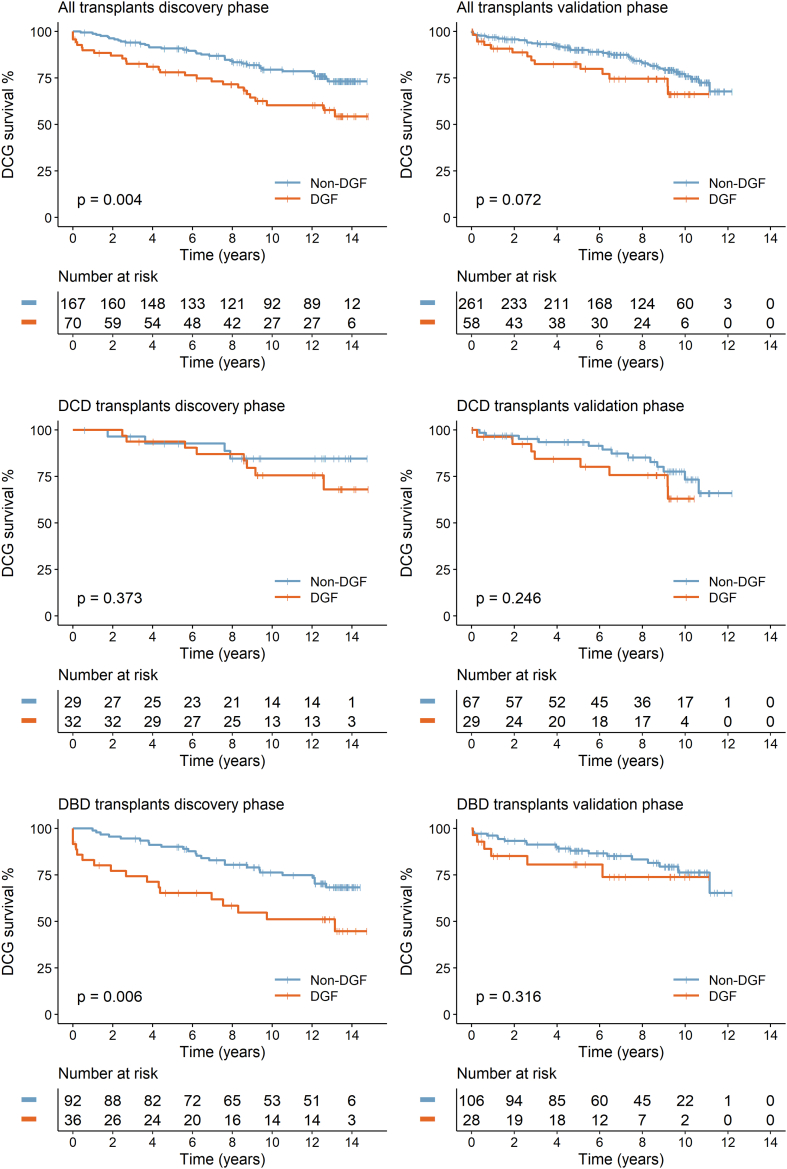
Kaplan-Meier curves for the discovery and validation phase cohorts showing death-censored graft survival by DGF status. For each phase of the study, results are shown for all transplants, DCD and DBD donor transplants. *P*-values shown are based on log-rank tests. DBD, donation after brain death; DCD, donation after circulatory death; DGF, delayed graft function.

Key patient characteristics are summarized in Table 1 with 79.7% of the transplants being DDKTs. Of these, 67.7% were donation after brain death (DBD). DGF was diagnosed in 29.5% of patients (70 events), with significant associations with donor type (28.1% of DBD vs. 52.5% of DCD transplants), time on dialysis, recipient ethnicity, human leukocyte antigen mismatch and lower eGFR at 1 year (Table 2). Median follow-up was 13.28 (IQR: 12.4–13.8) years with 64 patients classified as graft failure during follow-up. Five-year DCGS was 89.6% for living donor transplants and 86.5% for DDKTs with comparable figures for 10-year DCGS of 78.9% and 72.6%, respectively. DCD transplants had significantly better 5-year DCGS than DBD (93.3% vs. 83.3% respectively; *P* = 0.032) although not significant at 10 years (79.5% DCD vs. 69.5% DBD, *P* = 0.155). Other factors associated with outcomes included recipient age, CIT, and DGF status (Figure 2, Supplementary Figure S1) with 5-year DCGS of 79.4% in patients with DGF compared with 90.9% in patients with no DGF (Table 2, Figure 2).

**Table 1:** Key patient characteristics by donor type in discovery and validation phases

		Discovery phase				Validation phase				*P*-value (discovery vs. validation phase)			
		All (*n* = 237)	DBD (*n* = 128)	DCD (*n* = 61)	LD (*n* = 48)	All (*n* = 319)	DBD (*n* = 134)	DCD (*n* = 96)	LD (*n* = 89)	All	DBD	DCD	LD
Recipient age	Yrs	47.0 (16.0, 78.0)	49.0 (16.0, 75.0)	48.0 (19.0, 78.0)	36.0 (19.0, 65.0)	52.0 (19.0, 80.0)	48.5 (19.0, 80.0)	60.0 (23.0, 80.0)	47.0 (22.0, 74.0)	< 0.001	0.437	< 0.001	0.002
Recipient gender	Female	86 (36.3%)	55 (43.0%)	18 (29.5%)	13 (27.1%)	118 (37.0%)	51 (38.1%)	32 (33.3%)	35 (39.3%)	0.865	0.418	0.616	0.152
	Male	151 (63.7%)	73 (57.0%)	43 (70.5%)	35 (72.9%)	201 (63.0%)	83 (61.9%)	64 (66.7%)	54 (60.7%)				
Recipient ethnicity	White	185 (78.1%)	93 (72.7%)	50 (82.0%)	42 (87.5%)	289 (90.6%)	122 (91.0%)	84 (87.5%)	83 (93.3%)	< 0.001	0.001	0.343	0.204
	Asian	28 (11.8%)	20 (15.6%)	5 (8.2%)	3 (6.2%)	17 (5.3%)	5 (3.7%)	9 (9.4%)	3 (3.4%)				
	Black	6 (2.5%)	3 (2.3%)	3 (4.9%)	0 (0.0%)	6 (1.9%)	2 (1.5%)	2 (2.1%)	2 (2.2%)				
	Other	18 (7.6%)	12 (9.4%)	3 (4.9%)	3 (6.2%)	7 (2.2%)	5 (3.7%)	1 (1.0%)	1 (1.1%)				
Transplant type	DBD	128 (54.0%)	128 (100.0%)	(-)	(-)	134 (42.0%)	134 (100.0%)	(-)	(-)	0.016	(-)	(-)	(-)
	DCD	61 (25.7%)	(-)	61 (100.0%)	(-)	96 (30.1%)	(-)	96 (100.0%)	(-)				
	LD	48 (20.3%)	(-)	(-)	48 (100.0%)	89 (27.9%)	(-)	(-)	89 (100.0%)				
ESKD cause	CP	33 (13.9%)	17 (13.3%)	9 (14.8%)	7 (14.6%)	31 (9.7%)	11 (8.2%)	9 (9.4%)	11 (12.4%)	0.007	0.039	0.228	0.883
	Diabetes	23 (9.7%)	14 (10.9%)	5 (8.2%)	4 (8.3%)	19 (6.0%)	8 (6.0%)	6 (6.2%)	5 (5.6%)				
	GN	78 (32.9%)	37 (28.9%)	25 (41.0%)	16 (33.3%)	84 (26.3%)	30 (22.4%)	25 (26.0%)	29 (32.6%)				
	HTN	20 (8.4%)	13 (10.2%)	3 (4.9%)	4 (8.3%)	35 (11.0%)	14 (10.4%)	12 (12.5%)	9 (10.1%)				
	Inherited	31 (13.1%)	15 (11.7%)	8 (13.1%)	8 (16.7%)	77 (24.1%)	34 (25.4%)	20 (20.8%)	23 (25.8%)				
	Other	19 (8.0%)	11 (8.6%)	4 (6.6%)	4 (8.3%)	35 (11.0%)	19 (14.2%)	10 (10.4%)	6 (6.7%)				
	Unknown	33 (13.9%)	21 (16.4%)	7 (11.5%)	5 (10.4%)	38 (11.9%)	18 (13.4%)	14 (14.6%)	6 (6.7%)				
Previous transplants	0	205 (87.2%)	108 (85.0%)	55 (90.2%)	42 (89.4%)	260 (83.9%)	108 (83.7%)	83 (89.2%)	69 (78.4%)	0.272	0.771	0.855	0.113
	> 0	30 (12.8%)	19 (15.0%)	6 (9.8%)	5 (10.6%)	50 (16.1%)	21 (16.3%)	10 (10.8%)	19 (21.6%)				
	Missing	2	1	0	1	9	5	3	1				
Preemptive	No	218 (92.4%)	123 (96.1%)	58 (95.1%)	37 (78.7%)	231 (72.4%)	106 (79.1%)	75 (78.1%)	50 (56.2%)	< 0.001	< 0.001	0.004	0.009
	Yes	18 (7.6%)	5 (3.9%)	3 (4.9%)	10 (21.3%)	88 (27.6%)	28 (20.9%)	21 (21.9%)	39 (43.8%)				
	Missing	1	0	0	1	0	0	0	0				
Time on dialysis	Mths	30.4 (0.4, 192.0)	31.2 (3.9, 192.0)	37.1 (7.0, 164.7)	22.0 (0.4, 154.1)	33.0 (1.0, 495.0)	43.0 (3.0, 495.0)	24.0 (1.0, 281.0)	25.0 (1.0, 233.0)	0.998	0.012	0.003	0.523
	Missing	18	11	3	4	5	2	2	1				
Induction	Alem	13 (5.5%)	3 (2.3%)	8 (13.1%)	2 (4.2%)	172 (56.0%)	54 (42.5%)	67 (72.0%)	51 (58.6%)	< 0.001	< 0.001	< 0.001	< 0.001
	Bas	224 (94.5%)	125 (97.7%)	53 (86.9%)	46 (95.8%)	135 (44.0%)	73 (57.5%)	26 (28.0%)	36 (41.4%)				
	Missing	0	0	0	0	12	7	3	2				
Maintenance steroids	No	206 (86.9%)	115 (89.8%)	54 (88.5%)	37 (77.1%)	182 (57.1%)	60 (44.8%)	72 (75.0%)	50 (56.2%)	< 0.001	< 0.001	0.038	0.015
	Yes	31 (13.1%)	13 (10.2%)	7 (11.5%)	11 (22.9%)	137 (42.9%)	74 (55.2%)	24 (25.0%)	39 (43.8%)				
Total HLA mismatch	0–2	134 (56.5%)	94 (73.4%)	17 (27.9%)	23 (47.9%)	108 (34.1%)	53 (40.2%)	18 (18.8%)	37 (41.6%)	< 0.001	< 0.001	0.181	0.475
	3+	103 (43.5%)	34 (26.6%)	44 (72.1%)	25 (52.1%)	209 (65.9%)	79 (59.8%)	78 (81.2%)	52 (58.4%)				
	Missing	0	0	0	0	2	2	0	0				
CIT	Hr:mins	15:29 (0:29, 45:23)	17:34 (7:52, 45:23)	15:10 (8:38, 23:30)	1:31 (0:29, 16:26)	12:42 (0:25, 40:53)	15:4 (4:42, 40:53)	14:12 (7:46, 23:0)	3:43 (0:25, 16:9)	< 0.001	< 0.001	0.118	< 0.001
	Missing	0	0	0	0	5	2	1	2				
WIT	Mins	37.0 (12.0, 110.0)	35.0 (22.0, 57.0)	51.0 (17.0, 110.0)	32.5 (12.0, 80.0)	40.0 (2.0, 126.0)	36.0 (20.0, 100.0)	52.5 (18.0, 126.0)	32.0 (2.0, 69.0)	0.135	0.112	0.845	0.571
	Missing	0	0	0	0	17	11	2	4				
DGF status^a^	Non-DGF	167 (70.5%)	92 (71.9%)	29 (47.5%)	46 (95.8%)	261 (81.8%)	106 (79.1%)	67 (69.8%)	88 (98.9%)	0.002	0.173	0.005	0.246
	DGF	70 (29.5%)	36 (28.1%)	32 (52.5%)	2 (4.2%)	58 (18.2%)	28 (20.9%)	29 (30.2%)	1 (1.1%)				
eGFR (1 yr)		47.0 (10.0, 89.0)	46.0 (13.0, 89.0)	45.5 (10.0, 81.0)	53.0 (25.0, 73.0)	46.0 (6.0, 139.0)	46.0 (6.0, 139.0)	41.0 (11.0, 80.0)	51.0 (12.0, 105.0)	0.595	0.341	0.449	0.853
	Missing	13	10	0	3	47	18	16	13				
Overall patient survival	5 yrs	215 (90.7%)	114 (89.1%)	55 (90.2%)	46 (95.8%)	291 (91.2%)	123 (91.8%)	85 (88.5%)	83 (93.3%)	0.837	0.452	0.75	0.54
	10 yrs	181 (76.4%)	93 (72.7%)	47 (77.0%)	41 (85.4%)	258 (80.9%)	109 (81.3%)	74 (77.1%)	75 (84.3%)	0.197	0.094	0.996	0.859
	End of f/up	167 (70.5%)	84 (65.6%)	44 (72.1%)	39 (81.2%)	254 (79.6%)	106 (79.1%)	73 (76.0%)	75 (84.3%)	0.013	0.015	0.584	0.652
Death-censored return to dialysis	5 yrs	30 (12.7%)	21 (16.4%)	4 (6.6%)	5 (10.4%)	33 (10.3%)	17 (12.7%)	8 (8.3%)	8 (9.0%)	0.395	0.393	0.683	0.786
	10 yrs	56 (23.6%)	36 (28.1%)	11 (18.0%)	9 (18.8%)	58 (18.2%)	24 (17.9%)	20 (20.8%)	14 (15.7%)	0.116	0.049	0.667	0.652
	End of f/up	64 (27.0%)	42 (32.8%)	12 (19.7%)	10 (20.8%)	61 (19.1%)	25 (18.7%)	21 (21.9%)	15 (16.9%)	0.028	0.007	0.741	0.472

Alem, alemtuzumab; Bas, basiliximab; CIT, cold ischemic time; CP, chronic pyelonephritis; DBD, donation after brain death; DCD, donation after circulatory death (with all being controlled i.e., type III DCD donors); DGF, delayed graft function; eGFR, estimated glomerular filtration rate; ESKD, end stage kidney disease; f/up, follow-up.

GN, glomerulonephritis; HLA, human leukocyte antigen; HTN, hypertension; IQR, interquartile range; LD, live donor transplant; WIT, warm ischemic time.

The *P*-values relate to the comparison between the discovery and validation phase for each characteristic for each donor type using the Wilcoxon rank-sum test or Kruskal-Wallis rank test for continuous variables and the chi-square test for categorical variables.

For continuous variables, values in the table represent median (range), for categorical variables values in the table represent *n* (%)

a The number for DGF includes 3 primary non-function (PNF) grafts in the discovery phase.

**Table 2:** Deceased donor kidney transplants in the discovery and validation patient cohorts with clinical and demographic characteristics compared by DGF status

		Discovery phase (*n* = 189)			Validation phase (*n* = 230)		
		Non-DGF (*N* = 121)	DGF (*N* = 68)	*P*-value	Non-DGF (*N* = 173)	DGF (*N* = 57)	*P*-value
Recipient age (yrs)	Median (Range)	47.0 (16.0, 78.0)	53.5 (22.0, 75.0)	0.056	54.0 (19.0, 80.0)	53.0 (24.0, 77.0)	0.910
Recipient gender	Female	49 (40.5%)	24 (35.3%)	0.481	66 (38.2%)	17 (29.8%)	0.256
	Male	72 (59.5%)	44 (64.7%)		107 (61.8%)	40 (70.2%)	
Recipient ethnicity	White	95 (78.5%)	48 (70.6%)	0.040	156 (90.2%)	50 (87.7%)	0.634
	Asian	10 (8.3%)	15 (22.1%)		10 (5.8%)	4 (7.0%)	
	Black	4 (3.3%)	2 (2.9%)		2 (1.2%)	2 (3.5%)	
	Other	12 (9.9%)	3 (4.4%)		5 (2.9%)	1 (1.8%)	
Transplant type	DBD	92 (76.0%)	36 (52.9%)	0.001	106 (61.3%)	28 (49.1%)	0.107
	DCD	29 (24.0%)	32 (47.1%)		67 (38.7%)	29 (50.9%)	
ESKD cause	Chronic pyelonephritis	18 (14.9%)	8 (11.8%)	0.900	13 (7.5%)	7 (12.3%)	0.188
	Diabetes	11 (9.1%)	8 (11.8%)		7 (4.0%)	7 (12.3%)	
	Glomerulonephritis	40 (33.1%)	22 (32.4%)		44 (25.4%)	11 (19.3%)	
	Hypertension	12 (9.9%)	4 (5.9%)		21 (12.1%)	5 (8.8%)	
	Inherited	13 (10.7%)	10 (14.7%)		44 (25.4%)	10 (17.5%)	
	Other	10 (8.3%)	5 (7.4%)		20 (11.6%)	9 (15.8%)	
	Unknown	17 (14.0%)	11 (16.2%)		24 (13.9%)	8 (14.0%)	
Previous transplants	0	107 (89.2%)	56 (82.4%)	0.186	146 (87.4%)	45 (81.8%)	0.298
	> 0	13 (10.8%)	12 (17.6%)		21 (12.6%)	10 (8.2%)	
	Missing	1	0		6	2	
Pre-emptive	No	114 (94.2%)	67 (98.5%)	0.157	127 (73.4%)	54 (94.7%)	< 0.001
	Yes	7 (5.8%)	1 (1.5%)		46 (26.6%)	3 (5.3%)	
Time on dialysis (mths)	Median (Range)	29.2 (3.9, 192.0)	41.8 (10.0, 178.2)	0.017	30.0 (1.0, 495.0)	38.0 (1.0, 299.0)	0.158
	Missing	11	3		1	3	
Induction	Alemtuzumab	7 (5.8%)	4 (5.9%)	0.978	90 (53.9%)	31 (58.5%)	0.558
	Basiliximab	114 (94.2%)	64 (94.1%)		77 (46.1%)	22 (41.5%)	
	Missing	0	0		6	4	
Maintenance steroids	No	110 (90.9%)	59 (86.8%)	0.374	98 (56.6%)	34 (59.6%)	0.691
	Yes	11 (9.1%)	9 (13.2%)		75 (43.4%)	23 (40.4%)	
Total HLA mismatch	0–2	79 (65.3%)	32 (47.1%)	0.015	59 (34.5%)	12 (21.1%)	0.058
	3+	42 (34.7%)	36 (52.9%)		112 (65.5%)	45 (78.9%)	
	Missing	0	0		2	0	
CIT (h:min) DBD transplants	Median (Range)	17:42 (7:52, 45:23)	16:47 (13:0, 28:30)	0.637	14:42 (4:42, 40:53)	15:51 (7:0, 27:30)	0.366
	Missing	0	0		2	0	
CIT (h:min) DCD transplants	Median (Range)	14:21 (8:38, 20:50)	15:55 (10:36, 23:30)	0.061	13:49 (8:34, 23:0)	15:25 (7:46, 20:45)	0.254
	Missing	0	0		1	0	
WIT (min) DBD transplants	Median (Range)	35.0 (22.0, 57.0)	36.0 (25.0, 55.0)	0.092	35.0 (20.0, 100.0)	40.0 (24.0, 63.0)	0.036
	Missing	0	0		8	3	
WIT (min) DCD transplants	Median (Range)	50.0 (17.0, 84.0)	55.5 (18.0, 110.0)	0.161	52.0 (18.0, 126.0)	54.5 (33.0, 112.0)	0.295
	Missing	0	0		1	1	
Patient status at 1 yr	Alive with functioning graft	119 (98.3%)	61 (89.7%)	0.007	165 (95.4%)	50 (87.7%)	0.042
	Deceased and/or graft failed	2 (1.7%)	7 (10.3%)		8 (4.6%)	7 (12.3%)	
eGFR at 1 yr (ml/min per 1.73 m^2^)	Median (Range)	47.0 (13.0, 79.0)	37.5 (10.0, 89.0)	0.004	44.0 (11.0, 139.0)	41.0 (6.0, 100.0)	0.681
	Missing	9	1		23	11	
Overall patient survival	5 yrs	111 (91.7%)	58 (85.3%)	0.167	159 (91.9%)	49 (86.0%)	0.186
	10 yrs	91 (75.2%)	49 (72.1%)	0.636	141 (81.5%)	42 (73.7%)	0.204
	End of follow-up	84 (69.4%)	44 (64.7%)	0.506	137 (79.2%)	42 (73.7%)	0.385
Death-censored return to dialysis	5 yrs	11 (9.1%)	14 (20.6%)	0.025	16 (9.2%)	9 (15.8%)	0.169
	10 yrs	24 (19.8%)	23 (33.8%)	0.033	30 (17.3%)	14 (24.6%)	0.229
	End of follow-up	29 (24.0%)	25 (363.8%)	0.062	32 (18.5%)	14 (24.6%)	0.321
	Missing at end of follow-up	0	3		2	1	

CIT, cold ischemic time; DBD, donation after brain death; DCD, donation after circulatory death (with all being controlled i.e., type III DCD donors); DGF, delayed graft function; eGFR, estimated glomerular filtration rate; ESKD, end stage kidney disease; HLA, human leukocyte antigen; IQR, interquartile range; LD, live donor; WIT, warm ischemic time.

For continuous variables, values in the table represent median (range), for categorical variables values in the table represent *n* (%).

The *P*-values relate to the comparison between non-DGF and DGF in each phase using the Wilcoxon rank-sum test or Kruskal-Wallis rank test for continuous variables and the chi-square test for categorical variables.

#### Biomarker Analysis

**Figure 3:**
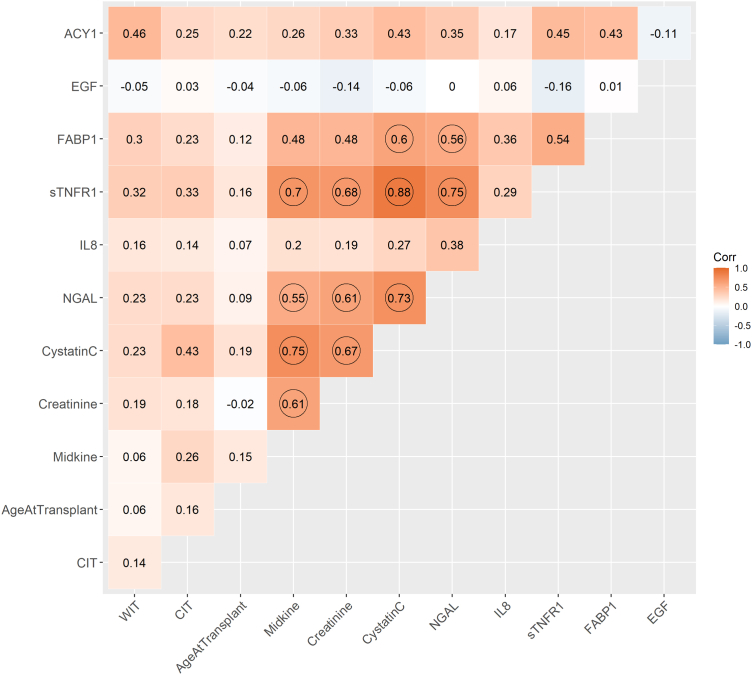
Correlation matrix of biomarkers measured in the discovery phase (posttransplant serum samples from days 1–3) and continuous clinical variables, considered for inclusion in the linear predictor. Numbers shown represent Spearman’s Rank correlation coefficients, with correlation ≥ 0.55 circled. ACY1, aminoacylase-1; CIT, cold ischemia time; EGF, epidermal growth factor; FABP1, fatty acid binding protein 1; IL-8, interleukin 8; NGAL, neutrophil gelatinase-associated lipocalin; sTNFR1, soluble tumor necrosis factor receptor-1; WIT, warm ischemic time.

Assay performance details are provided in Supplementary Table S2. Excellent correlation was observed (*n* = 377, r_S_ = 0.95, *P* < 0.001) between our original ACY1 enzyme-linked immunosorbent assay^26^ and the prototype ACY1 biochip, with absolute values differing due to recalibration (Supplementary Figure S2). Biomarker distributions are shown in Supplementary Figure S3 with Cr, CysC, midkine, sTNFR1, and NGAL showing strongest correlations (Figure 3). Most (except EGF) showed significant associations with factors including donor type, preemptive transplant, and particularly DGF status, CIT, and warm ischemic time (Supplementary Table S3).

**Figure 4:**
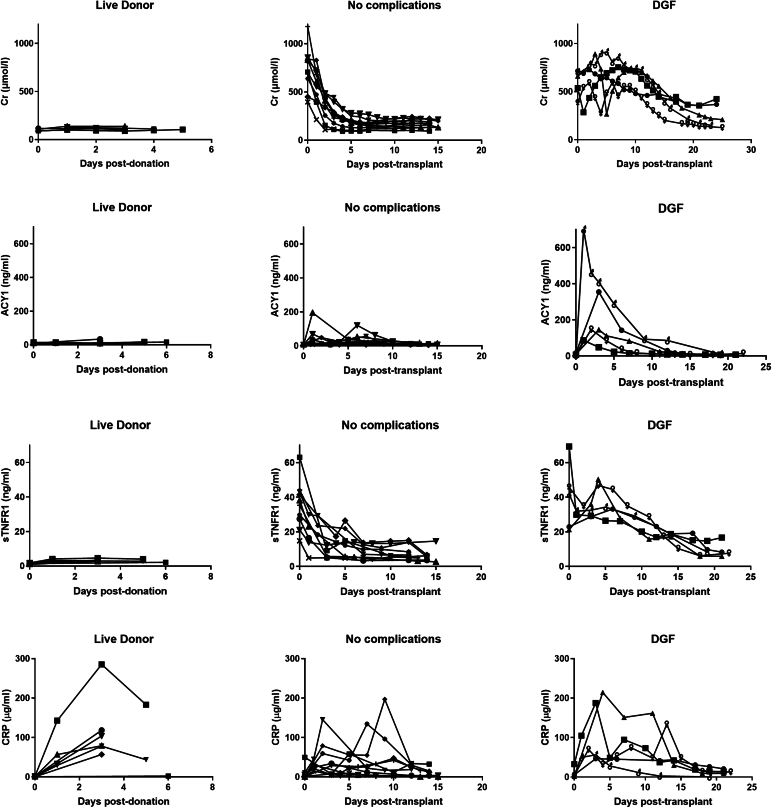
Baseline and profiles of serum biomarkers selected to illustrate the varying patterns observed. Profiles for creatinine, aminoacylase-1, soluble tumor necrosis factor receptor-1, and C-reactive protein are shown for live donors prior to and after kidney donation (*n* = 7; aged 32–62 years), patients with end-stage kidney disease prior to and following kidney transplantation with either no acute complications (*n* = 10), or where DGF was diagnosed (*n* = 5). Day 0 represents the day of kidney donation (live donors) or kidney reperfusion following transplant, with day 0 results from samples taken on the day prior to the event or on the previous day in 5 cases. DGF, delayed graft function.

In patients with ESKD prior to transplantation, most biomarkers were elevated compared with living donors prior to donation. This included Cr, sTNFR1, midkine, fatty acid binding protein 1, and CysC, which declined posttransplant although more slowly with DGF. In contrast, C-reactive protein, interleukin-8, and EGF were mainly affected by surgery, increasing pos-transplant, and in living donors postnephrectomy, before declining. ACY1 concentrations uniquely were low pretransplantation, increasing posttransplantation particularly with DGF, before declining in the first week posttransplant, with little change seen in living donors. Illustrative examples are shown in Figure 4.

#### Initial Biomarker Shortlisting for Linear Predictor Models and the RTP Biochip

ACY1 and CysC were consistently selected by penalized LASSO regression for prediction of DGF and DCGS, with recipient age and DCD donor type as the most promising clinical variables (Supplementary Table S4). Midkine and sTNFR1 were also shortlisted for inclusion in the final model development based on these initial results and other considerations, including balancing relative performances across both outcomes, multiplexing compatibility, assay ranges and biological interest. Serum Cr was for measured as the gold standard comparator.

#### Prediction of DGF

Univariable analysis identified recipient age, donor type, warm ischemic time, and all biomarkers, except EGF and interleukin-8, as significantly associated with DGF for all transplants and DDKTs (Supplementary Table S5).

**Figure 5:**
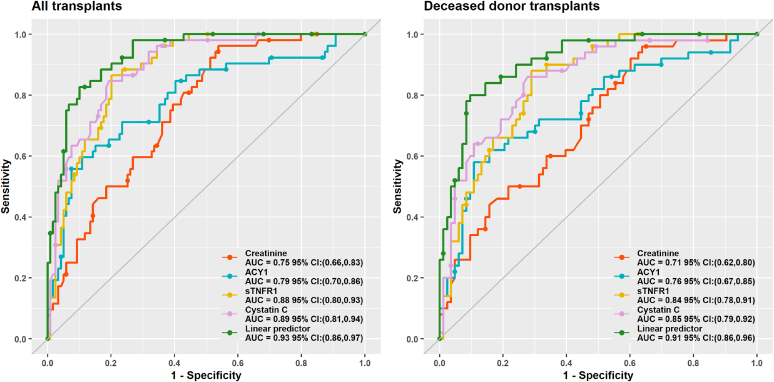
Receiver operating characteristic curves for serum ACY1, Cr, CysC, and sTNFR1 together with the linear predictor derived from the combination of ACY1, CysC, and sTNFR1 for the prediction of delayed graft function on days 1 to 2 posttransplant in the discovery phase (*n* = 173). Results are shown for all transplants and deceased donor transplants. ACY1, aminoacylase-1; AUC, area under the curve, Cr, creatinine; CysC, cystatin C; DGF, delayed graft function; sTNFR1, soluble tumor necrosis factor receptor-1.

The final linear predictor for all transplants was: linear predictor = −6.56 + (log(ACY1)∗0.41) + (log(CysC)∗ 2.19) + (log(sTNFR1)∗0.55). This was also used for the DDKTs alone because the predictor developed using that dataset was almost identical. The AUROC of 0.93 (95% CI: 0.86–0.97) confirmed our original results^26^ and was significantly higher than the AUROC for Cr (0.75; 95% CI: 0.66–0.83), and the component biomarkers (0.79–0.89; Figure 5, Table 3). In particular, the linear predictor had greater specificity and positive predictive value compared with individual biomarkers (Table 3), although ACY1 showed similar specificity but much lower sensitivity. Results were slightly lower for DDKTs. Although not selected in the linear predictor, the AUROCs for midkine (0.76; 95%CI: 0.69–0.84), NGAL (0.82; 95%CI: 0.76–0.88) and fatty acid binding protein 1 (0.80; 95% CI: 0.73–0.87) were also high.

**Table 3:** AUCs, sensitivity, specificity and predictive values for the individual biomarkers and the linear predictor for prediction of DGF in the discovery phase for a). all transplants and b). deceased donor transplants only

Marker	AUC (95% CI)	Threshold	Sensitivity % (95% CI)	Specificity % (95% CI)	PPV (95% CI)	NPV (95% CI)	Youden index	TP	FN	TN	FP	AUC test
(a) All transplants												
Cr	0.75 (0.66, 0.83)	< −1.523	96.2 (90.9–100.0)	46.2 (37.3–55.2)	43.9 (34.8–53.0)	96.5 (91.7–100.0)	0.42	50	2	55	64	< 0.001
ACY1	0.79 (0.70, 0.86)	< −0.375	59.6 (46.3–73.0)	89.1 (83.5–94.7)	70.5 (57.0–83.9)	83.5 (77.0–89.9)	0.49	31	21	106	13	0.001
sTNFR1	0.88 (0.80, 0.93)	< −0.806	86.5 (77.3–95.8)	79.8 (72.6–87.0)	65.2 (54.0–76.5)	93.1 (88.2–98.0)	0.66	45	7	95	24	0.105
CysC	0.89 (0.81, 0.94)	< −0.609	84.6 (74.8–94.4)	80.7 (73.6–87.8)	65.7 (54.3–77.0)	92.3 (87.2–97.4)	0.65	44	8	96	23	0.195
Linear predictor	0.93 (0.86, 0.97)	< −0.391	82.7 (72.4–93.0)	89.9 (84.5–95.3)	78.2 (67.3–89.1)	92.2 (87.4–97.1)	0.73	43	9	107	12	(-)

(b) Deceased donor transplants												
Marker	AUC (95% CI)	Threshold	Sensitivity % (95% CI)	Specificity % (95% CI)	PPV (95% CI)	NPV (95% CI)	Youden index	TP	FN	TN	FP	AUC test
Cr	0.71 (0.62, 0.80)	< −1.523	96.0 (90.6–100.0)	36.1 (25.8–46.5)	47.5 (37.8–57.3)	93.8 (85.4–100.0)	0.32	48	2	30	53	< 0.001
ACY1	0.76 (0.67, 0.85)	< −0.375	62.0 (48.5–75.5)	84.3 (76.5–92.2)	70.5 (57.0–83.9)	78.7 (70.1–87.2)	0.46	31	19	70	13	< 0.001
sTNFR1	0.84 (0.78, 0.91)	< −0.806	88.0 (79.0–97.0)	71.1 (61.3–80.8)	64.7 (53.4–76.1)	90.8 (83.7–97.8)	0.59	44	6	59	24	0.002
CysC	0.85 (0.79, 0.92)	< −0.609	86.0 (76.4–95.6)	72.3 (62.7–81.9)	65.2 (53.7–76.7)	89.5 (82.2–96.9)	0.58	43	7	60	23	0.045
Linear predictor	0.91 (0.86, 0.96)	< −0.391	82.0 (71.3–92.7)	85.5 (78.0–93.1)	77.4 (66.1–88.6)	88.8 (81.8–95.7)	0.68	41	9	71	12	(-)

ACY1, aminoacylase-1; AUC, area under the curve; Cr, creatinine; CysC, cystatin C; FN, false negative; FP, false positives; NPV, negative predictive value; PPV, positive predictive value; sTNFR1, soluble tumor necrosis factor receptor-1; TN, true negative; TP, true positive.

The linear predictor is based on ACY1, sTNFR1, and CysC as measured in serum samples on days 1 to 2 post-renal transplant. The values for Cr are shown as the current gold standard biomarker used routinely to monitor patients.

#### Prognosis - DCGS

Univariable analysis showed recipient ethnicity, time on dialysis, maintenance steroids, NGAL, CysC, and Cr to be significantly associated with DCGS, with CIT just failing to achieve significance (Supplementary Table S6).

**Figure 6:**
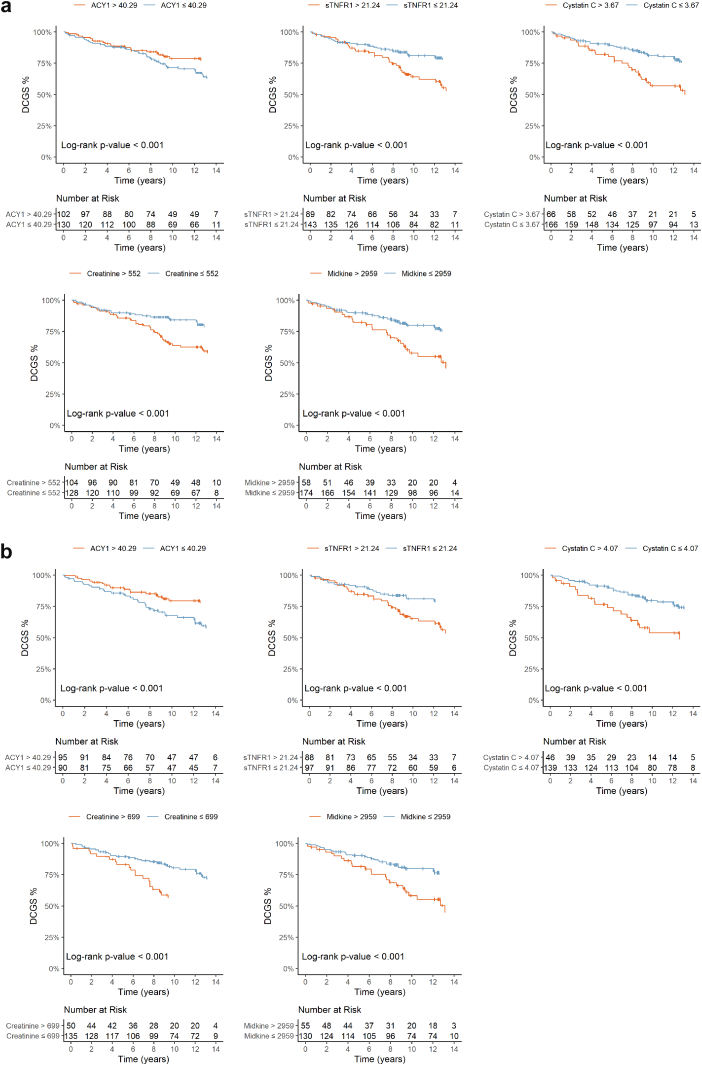
Kaplan-Meier curves for DCGS stratified by serum biomarkers on days 1 to 3 posttransplant in the discovery phase for (a) All transplant patients and (b) deceased donor transplants. ACY1, aminoacylase-1; DCGS, death-censored graft survival; sTNFR1, soluble tumor necrosis factor receptor-1.

The final linear predictors were: All transplants -> (log(ACY1)∗-0.06) + (log(CysC)∗0.44) + (log(midkine)∗0.01) + (age at transplant∗-0.01), and DDKTs only -> (log(ACY1)∗-0.04)+(log(CysC)∗0.15) + (log(midkine)∗0.01). However, k-statistics were only 0.55 (95% CI: 0.48–0.63) in all transplants and 0.52 (95% CI: 0.43–0.60) in DDKTs when adjusted for overfitting. Kaplan-Meier analysis of component biomarkers and sTNFR1 (as on the RTP biochip), showed significant associations with DCGS although varying in curve divergence timepoints, and with greater separation in DDKTs (Figure 6).

### Validation Phase

#### Patient Cohort

The validation cohort differed in increased recipient age, preemptive transplantation, induction therapy, maintenance steroids, shorter CIT, and increased human leukocyte antigen mismatch (Table 1). The proportion of living donor transplants overall was greater (27.9% vs. 20.3%) and of DCD transplants in the DDKTs (41.7% vs. 32.3%). The DGF rate was lower (18.2% vs. 29.5%) with 58 DGF events overall, mainly because of the lower DGF frequency within DDKTs (24.8% vs. 36% validation and discovery, respectively), particularly in DCD (30.2% vs. 52.5%), but also in DBD transplants (20.9% vs. 28.1%), and with preemptive transplantation being associated with lower DGF frequency (Table 2).

During follow-up (median: 9.03 years, IQR: 5.19–10.16), 61 graft failures occurred (Table 1). Except for DBD transplants, where 10-year DCGS was significantly higher in the validation phase (82.1% vs. 71.9%, *P* = 0.049), 1-year eGFR or 5- or 10-year DCGS within donor types or by DGF status were similar to those in the discovery phase (Tables 1 and 2, Figure 2). No significant differences in 5- or 10-year DCGS between DCD and DBD transplants were observed (DBD 86.5% vs. DCD 90.1%; *P* = 0.331; DBD 75.32% vs. DCD 70.2%, *P* = 0.514 respectively). Kaplan-Meier analysis showed significant associations of CIT, previous transplants, and recipient age with DCGS (Supplementary Figure S1).

#### RTP Biochip Development and Biomarker Analysis

The final RTP biochip used to analyze validation phase samples measured ACY1, CysC, and sTNFR1 (Supplementary Table S7) with midkine measured by enzyme-linked immunosorbent assay.

#### Prediction of DGF

**Figure 7:**
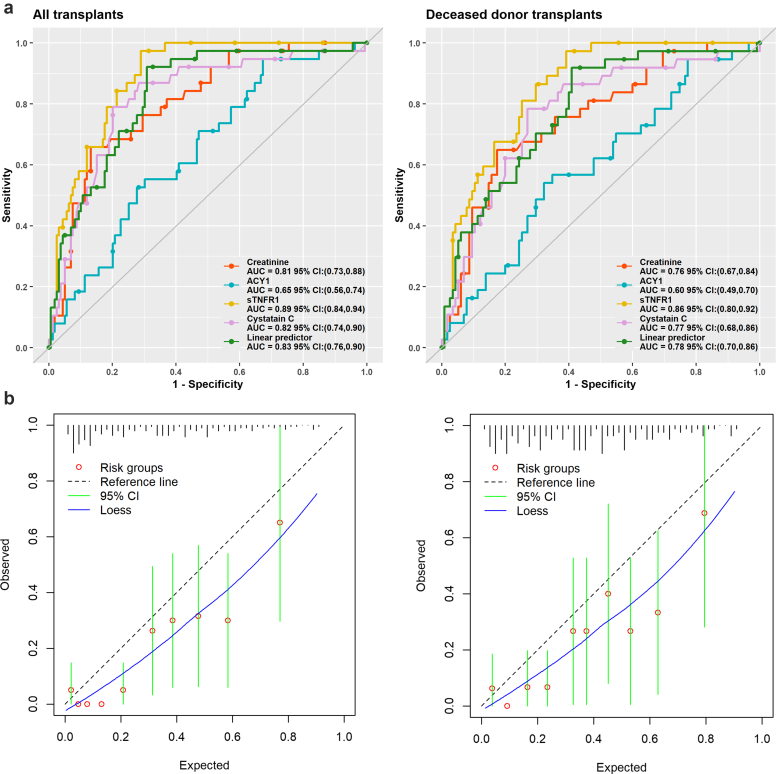
(a) Receiver operating characteristic curves for the linear predictors derived from the discovery phase and applied to data from the validation cohort for prediction of DGF for either all transplants or just deceased donor transplants. Receiver operating characteristic curves are also shown for ACY1, CysC, and sTNFR1 as the individual serum biomarker components of the linear predictor (days 1 or 2 posttransplant) and Cr as the gold standard. (b) Corresponding calibration plots for the linear predictors derived in the discovery phase data and applied to the validation phase data with slopes of 1.06 and 0.93 respectively. ACY1, aminoacylase-1; AUC, area under the curve; CysC, cystatin C; sTNFR1, soluble tumor necrosis factor receptor-1.

Applying the linear predictor to validation phase data achieved an AUROC of 0.83 (95% CI: 0.76–0.90) in all transplants (Table 4, Figure 7a). Calibration plots (Figure 7b) showed an expected/observed proportion of 1.57 with observed levels being consistently lower. Notably, sTNFR1 performed similarly (AUROC: 0.89) and significantly better than the linear predictor (*P* = 0.049) whereas ACY1 was markedly reduced (AUROC: 0.65). Similar trends were observed for DDKTs.

**Table 4:** Validation phase cohort AUCs, sensitivity, specificity and predictive values for the component individual biomarkers and linear predictors developed in the discovery phase for prediction of DGF in a). all transplants and b). deceased donor transplants only

Marker	AUC (95% CI)	Threshold	Sensitivity % (95% CI)	Specificity % (95% CI)	PPV (95% CI)	NPV (95% CI)	Youden index	TP	FN	TN	FP	AUC test
(a) All transplants												
Cr	0.81 (0.73, 0.88)	< −1.523	81.6 (69.2–93.9)	59.8 (52.1–67.4)	32.6 (23.2–42.1)	93.1 (88.2–98.0)	0.41	31	7	95	64	0.589
ACY1	0.65 (0.56, 0.74)	< −0.375	55.3 (39.5–71.1)	68.5 (61.3–75.8)	29.6 (19.0–40.2)	86.5 (80.5–92.5)	0.24	21	17	109	50	< 0.001
sTNFR1	0.89 (0.84, 0.94)	< −0.806	100.0 (100.0–100.0)	57.9 (50.2–65.5)	36.2 (27.0–45.4)	100.0 (100.0–100.0)	0.58	38	0	92	67	0.049
CysC	0.82 (0.74, 0.90)	< −0.609	63.2 (47.8–78.5)	84.3 (78.6–89.9)	49.0 (35.0–63.0)	90.5 (85.8–95.3)	0.47	24	14	134	25	0.59
Linear predictor	0.83 (0.76, 0.90)	< −0.391	71.0 (56.6–85.5)	76.1 (69.5–82.7)	41.5 (29.6–53.5)	91.7 (87.0–96.4)	0.47	27	11	121	38	(-)
(b) Deceased donor transplants												
Cr	0.76 (0.67, 0.84)	< −1.523	81.1 (68.5–93.7)	49.6 (40.4–58.7)	34.1 (24.2–44.0)	89.1 (81.4–96.7)	0.31	30	7	57	58	0.64
ACY1	0.60 (0.49, 0.70)	< −0.375	56.8 (40.8–72.7)	63.5 (54.7–72.3)	33.3 (21.7–45.0)	82.0 (74.0–90.0)	0.20	21	16	73	42	< 0.001
sTNFR1	0.86 (0.80, 0.92)	< −0.806	100.0 (100.0–100.0)	46.1 (37.0–55.2)	37.4 (27.8–46.9)	100.0 (100.0–100.0)	0.46	37	0	53	62	0.038
CysC	0.77 (0.68, 0.86)	< −0.609	62.2 (46.5–77.8)	79.1 (71.7–86.6)	48.9 (34.6–63.2)	86.7 (80.2–93.2)	0.41	23	14	91	24	0.66
Linear predictor	0.78 (0.70, 0.86)	< −0.391	70.3 (55.5–85.0)	67.8 (59.3–76.4)	41.3 (29.1–53.4)	87.6 (80.8–94.5)	0.38	26	11	78	37	(-)

ACY-1, aminoacylase-1; AUC, area under the curve; Cr, creatinine; CysC, cystatin C; FN, false negative; FP, false positives; NPV, negative predictive value; PPV, positive predictive value; sTNFR1, soluble tumor necrosis factor receptor-1; TN, true negative; TP, true positive.

The linear predictor is based on ACY1, sTNFR1, and CysC as measured in serum samples on days 1 to 2 post-renal transplant. The values for Cr are shown as the current gold standard biomarker used routinely to monitor patients.

#### Prognosis - DCGS

**Figure 8:**
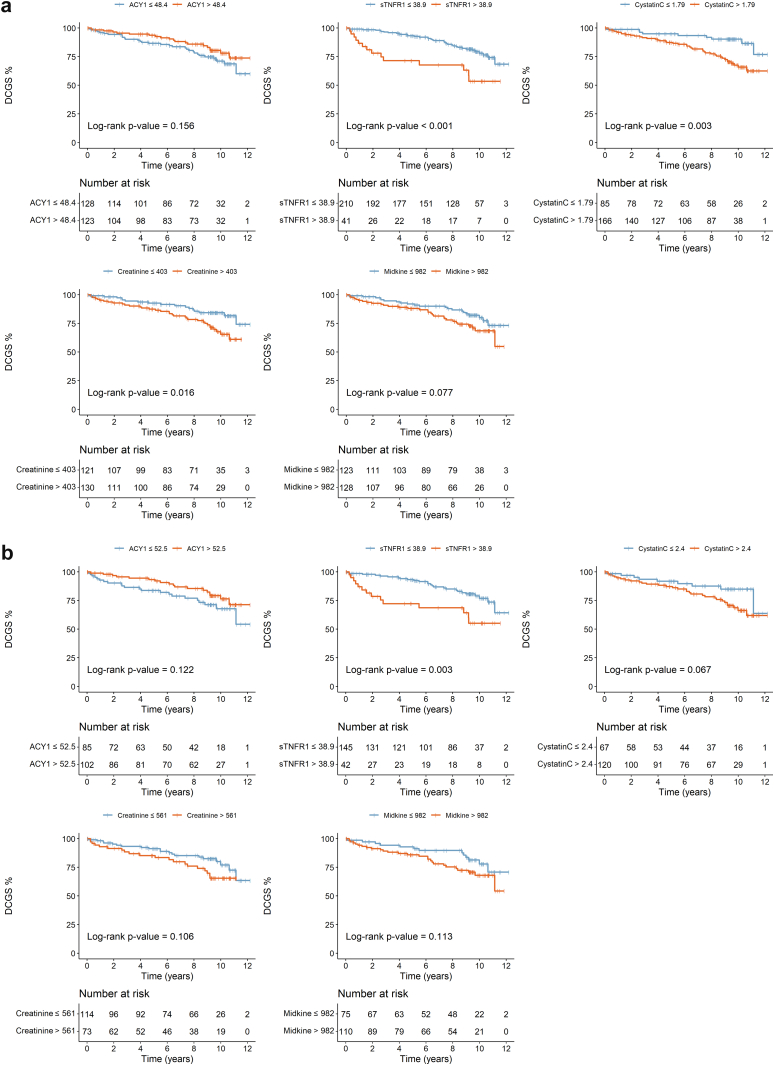
Kaplan-Meier curves for death-censored graft survival stratified by serum biomarkers on days 1 to 3 posttransplant in the validation phase for (a) all transplant patients and (b) deceased donor transplants. ACY1, aminoacylase-1; DCGS, death-censored graft survival; sTNFR1, soluble tumor necrosis factor receptor-1.

**Figure 9:**
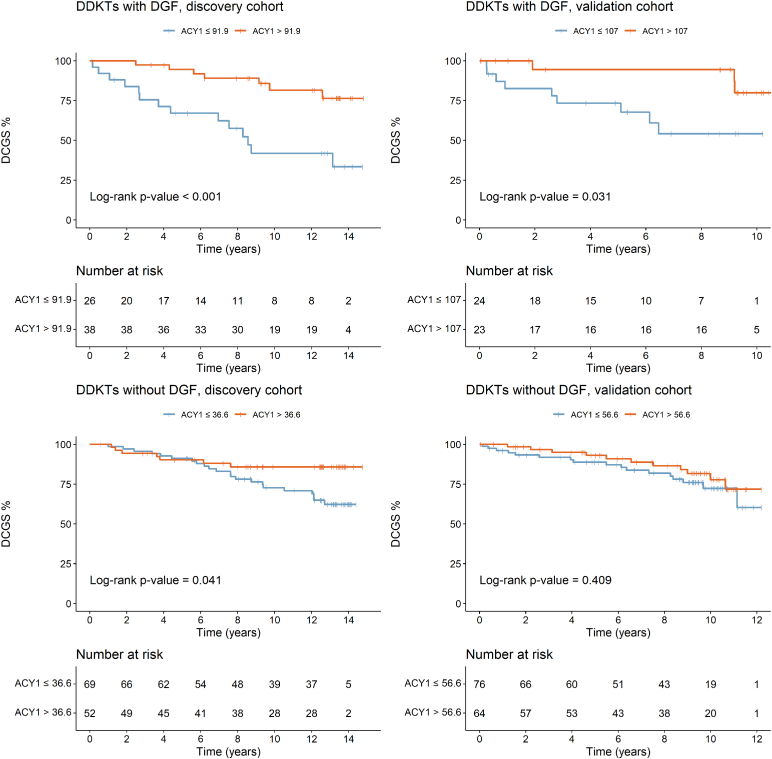
Kaplan-Meier curves for death-censored graft survival stratified by serum ACY1 on days 1 to 3 posttransplant for deceased donor transplants in either DGF or non-DGF subgroups in the discovery and validation cohorts. ACY1, aminoacylase-1; DGF, delayed graft function; DDKT, deceased donor kidney transplants.

Given the low k-statistics for the linear predictors, we focused on individual biomarkers. Considering all transplants, sTNFR1, CysC, and Cr still showed similar significant stratification (Figure 8a), and in the DDKTs (Figure 8b), only sTNFR1. Driven by our original findings,^26^ exploratory analysis of the DDKTs with DGF, recognizing the limited patient numbers and events, found prognostic stratification potential in all 5 biomarkers (Figure 9, Supplementary Figure S4) although only confirmed for ACY1 and CysC in the validation cohort.

### Further Exploratory Analyses

**Figure 10:**
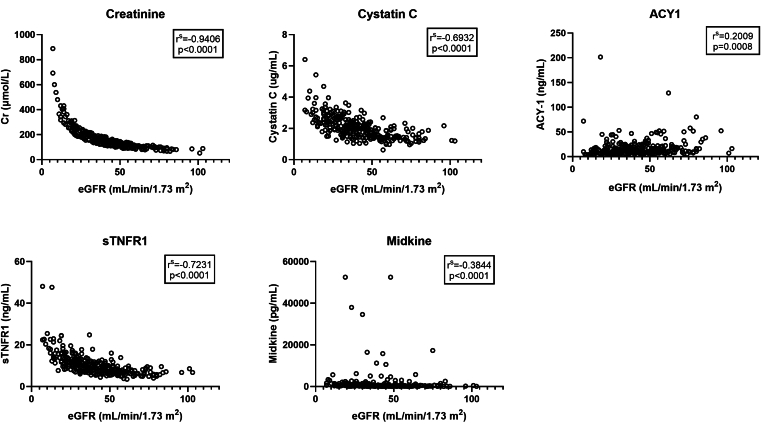
Correlation plots of the serum biomarkers Cr, CysC, ACY1, sTNFR1, and midkine with eGFR (*n* = 273 pairs) as determined in patients at 4 weeks post-transplant (+/−4 days) with significance testing using Spearman Rank. ACY1, aminoacylase-1; Cr, creatinine; CysC, cystatin C; eGFR, estimated glomerular filtration rate; sTNFR1, soluble tumor necrosis factor receptor-1.

At 4 weeks posttransplant, CysC, sTNFR1, and midkine (but not ACY1) were significantly negatively correlated with eGFR,^47^ comparable with Cr (Figure 10).

**Figure 11:**
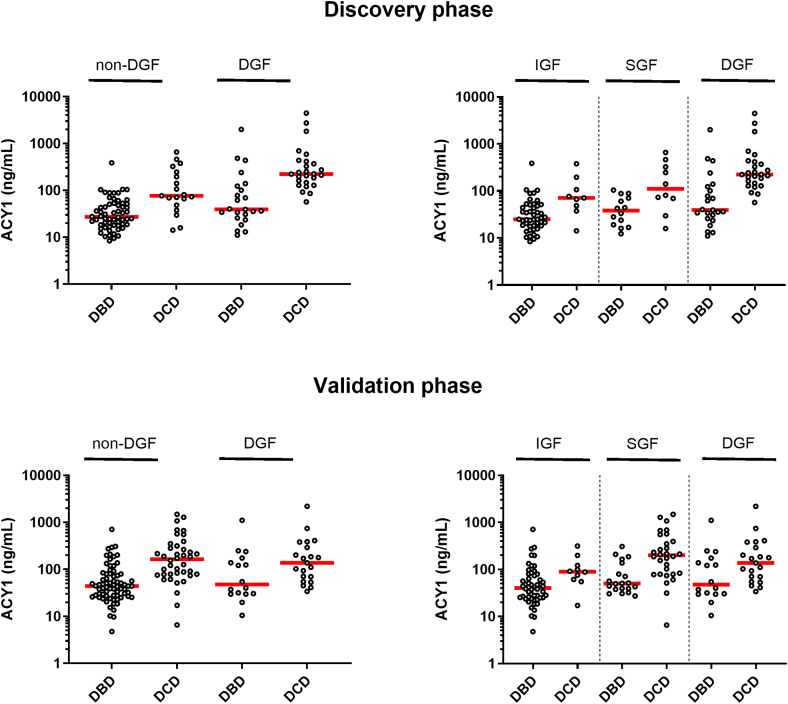
Serum biomarker distribution plots for ACY1 (log-scale) for the deceased donor transplants on days 1 or 2 posttransplant in each of the discovery and validation phases subdivided on the basis of non-DGF and DGF, or alternatively by IGF, SGF, or DGF. Horizontal red lines show the medians. ACY1, aminoacylase-1; DBD, donation after brain death; DCD, donation after circulatory death; DGF, delayed graft function; IGF, immediate graft function; SGF, slow graft function.

To explore possible changes in ACY1 distributions impacting on DGF prediction, applying the functional DGF concept to subdivide non-DGF into slow or immediate graft function (SGF, IGF), using day 5 serum Cr,^48^^,^^49^ ACY1 alone was found at significantly higher concentrations in DCD compared with DBD transplants within each functional DGF subgroup in both phases (Figure 11; IGF *P* = 0.0037 vs. 0.013; SGF *P* = 0.022 vs. < 0.0001; DGF *P* ≤ 0.0001 vs. *P* = 0.0374 discovery and validation phases, respectively). The proportions of living donor, DBD, and DCD transplants with IGF varied little across the discovery and validation phases (living donor: 93.5% vs. 92.1%, DBD: 54.9% vs. 56%, DCD: 19% vs. 16.8%, respectively). In marked contrast, in DCD transplants, SGF and DGF proportions reversed between discovery (25.9% SGF/55.1% DGF) and validation (52.6% SGF/30.5% DGF) phases. In parallel, median ACY1 concentrations decreased in the DCD DGF group from 223.4 to 137.4 ng/ml (*P* = 0.0306) and conversely, increased in the DCD SGF group from 110.8 to 199.6 ng/ml (NS; Figure 11). ACY-1 concentrations for SGF and DGF groups combined were unchanged between study phases (medians 217.3 ng/ml vs. 178.3 ng/ml; NS).

## Discussion

Biomarkers are increasingly needed in renal transplantation^24^ and the Kidney Disease: Improving Global Outcomes highlighted the need for accurate prediction of allograft failure^23^ although many prognostic studies have limitations.^50^ We describe several biomarkers with potential for prediction of DGF and outcome, and importantly also illustrating the impacts of clinical changes over time.

Predicting DGF is inherently challenging given its mechanistic heterogeneity and varying threshold for dialysis, with DGF rates varying from 3.2% to 63.3% in DDKTs across USA centers using 1 definition.^51^ The AUROC of 0.93 (0.83 in validation) for our DGF linear predictor based on ACY1, CysC and sTNFR1 combined, outperformed Cr and the most widely cited (although now unavailable online) tool for DDKTs (AUROC: 0.704) with 20 clinical variables.^7^ An alternative model limited to nonpreemptive and nonmachine perfused DDKTs included 5 clinical variables and had an AUROC of 0.73 in validation.^52^ The high NPV (90%–100%) for our linear predictor and component biomarkers (excluding ACY1) warrants exploration in larger studies for trial enrichment, or potential use in avoiding dialysis in modestly fluid-loaded patients with minimal urine output but not life-threatening features when functional transplant improvement was predicted.

The reduced performance of the DGF linear predictor may be due to underpowering with the historical cohort samples available for model development (70 DGF events equating to 4–5 events per variable), although bootstrapping mitigated overfitting. Using the powerROC tool,^46^ our validation cohort was adequate with 61 DGF events, although a minimum of 100 events has also been recommended.^53^ Since commencing this study, the failure of large numbers of multivariable predictive models because of inadequate-sized datasets or other design issues has been recognised.^54^ Moving away from “rules of thumb” and events per variable, recent guidance provides design considerations for model development and validation, considering context and required performance.^54^, ^55^, ^56^

For prognostic stratification or as surrogate end points to accelerate clinical trials, individual parameters such as proteinuria, and eGFR are inadequate.^23^^,^^57^ Reports of >30 different causes underlying graft loss,^58^ 8 different eGFR trajectories posttransplant, and 7 independent donor or recipient determinants,^59^ illustrate the need for multivariable models. The iBOX score integrating 8 immunological, histological, and functional variables,^60^ is the most extensively validated, with recent European Medicines Agency approval as a novel trial end point for long-term graft outcome. Our DCGS rates reflect national data^61^^,^^62^ and the similar or slightly better outcome for DCD compared with DBD transplants, despite the much higher DGF incidence, agrees with others.^12^^,^^14^^,^^63^, ^64^, ^65^, ^66^ However, our multivariable model showed inadequate performance in the discovery phase, potentially because of cohort size with only 64 graft failures. Individually, sTNFR1, CysC, and Cr were prognostic across both phases. Although very preliminary, the stratification of DDKTs affected by DGF by ACY1 and CysC in both the discovery and validation cohorts is promising, confirming our previous findings.^26^ These biomarkers should be explored further in combination with relevant clinical variables in much larger studies.

Importantly these biomarkers have biological plausibility. CysC is a marker of renal function and its DGF predictive potential has been confirmed.^67^^,^^68^ TNF is involved in inflammatory and stress response pathways and processes implicated in renal damage and fibrosis.^5^ TNF pathway activation has been linked to the potential resilience of DCD transplants to DGF and DGF duration.^13^^,^^31^ sTNFRs are inversely related to eGFR and prognostic for renal function decline^69^ but clearly complement CysC and Cr. ACY1 catalyzes hydrolysis of N-acetylated proteins, freeing up amino acids, particularly in renal proximal tubules.^70^ With little association with eGFR or inflammatory biomarkers, kinetics demonstrated likely production by the kidney during transplantation, also supported by ACY1 detection in perfusate fluid (unpublished data). ACY1 regulates sphingosine kinase-1 activity,^71^ implicated in the repair response to renal ischemia-reperfusion injury^72^ and kidney disease and the target of novel therapies.^73^

The striking donor type–specific ACY1 distributions confirms our previous findings.^26^ Reduced ACY1 concentrations in the DCD DGF subgroup during validation likely also contributed to the decreased ACY1 AUROC and the poorer performance of the linear predictor. Using functional DGF, now included as a potential end point in the US Food and Drug Agency guidance for evaluation of drugs for prevention of DGF^74^ and adopted in various ways in several studies,^8^^,^^18^, ^19^, ^20^, ^21^^,^^48^^,^^49^^,^^75^, ^76^, ^77^ we showed that DGF/SGF classifications were reversed across discovery and validation cohorts, particularly for DCD transplants. In parallel, the significantly higher ACY1 concentrations in DCD transplants compared with DBD were much less marked in the DGF group and more marked in the SGF group in the validation cohort. This, together with no change in the IGF proportion and ACY1 distributions being similar for DCD transplants across phases when grouped by either IGF or SGF/DGF, suggests a possible difference in decision to dialyze threshold (and hence DGF classification) over time, with increasing experience with DCD transplants although other clinical practice changes may also impact.

In DBD transplants, DGF has been associated with more marked effects on outcome^12^, ^13^, ^14^^,^^64^ although shorter DGF-associated dialysis periods^9^^,^^13^ and higher post-DGF eGFR^13^ suggest a less severe phenotype. The greater resilience of DCD transplants to DGF may reflect upregulated tissue repair and proliferation and downregulation of proinflammatory pathways compared with DBD kidneys.^13^ Higher ACY1 concentrations in DCD transplants, particularly with SGF/DGF, aligns with this and possibly reflects the greater tissue repair response. Conversely, lower ACY1 concentrations in some transplant patients with DGF may indicate less repair response and an association with poorer outcome, as we found.

In terms of further clinical impacts, adult kidney-only DCD transplants in the UK increased from 143 (12.5% of DDKTs) in 2004 and 2005 to 851 (42.9% of DDKTs) in 2015 and 2016,^78^^,^^79^ as reflected across our cohorts. The marked decrease in CIT between phases reflects UK trends^78^^,^^79^ with increases in preemptive transplants, human leukocyte antigen mismatch, maintenance steroids, and change from basixilimab to alemtuzumab observed. The higher incidence of DGF in DCD transplants is widely recognized^3^^,^^7^^,^^9^^,^^11^, ^12^, ^13^, ^14^^,^^63^; however, our observation of this almost halving during our study timeframe is novel, leading to fewer DGF events than anticipated. It is supported by reported DGF rates of 50% in UK DCD transplants in 2000 to 2007,^11^ similar to our discovery cohort, and a steady decline from 52% to 30% over 2006 to 2016 from reexamination of UK registry data^17^ (B Phillips-personal communication). This is despite increased donor and recipient complexity. These differences between historical and contemporary cohorts, with some being reported previously,^63^, ^80^ illustrate the difficulties in data comparison and study design. Clinical practice changes were reflected in the updated predictive model developed by Irish, with weightings of many factors changing between 2003 and 2010.^7^

Multicenter biomarker studies pose major logistical challenges and minimizing preanalytical confounding through high quality samples is arguably as important as ensuring adequate patient numbers. Strengths of our study include standardized sample and clinical data collection, long-term follow-up, and a multicenter independent validation cohort. Limitations of this study include wide (24-hour) sample windows, moderate cohort sizes, limited availability of donor-related variables and recipient urinary output for the discovery cohort, and the impact of clinical changes across the cohorts.

In conclusion, several biomarkers have potential for predicting DGF and/or prognosis but need studies of sufficient scale and design^24^^,^^50^^,^^54^, ^55^, ^56^ to allow evaluation in combination with relevant donor and recipient factors in different DDKT types and functional DGF categories. This may allow delineation of specific phenotypes reflecting pathophysiological processes during procurement and transplantation, contributing to evolving potential surrogate endpoints or trial enrichment strategies.

## Disclosure

PJS held the NIHR Programme grant funding which contributed to funding sample biobanking at clinical sites. REB was a named grant holder jointly with Randox Laboratories on the Innovate UK funded aspects of the study and with the earlier funding from the Department of Health, and the University of Leeds received patent funding from Randox Laboratories. MJK, JW, DM, HW, and MR are or were employees of Randox Laboratories Ltd but hold no shares in the company. PF is the Managing Director and owner of Randox Laboratories Ltd (a privately owned company). SB is a member of the Advisory Board and received speaker renumeration from Astellas, CSL Vifor, and GSK, DR has received speaker honoraria from Astellas and meeting attendance costs from Sandoz. MWS received consulting fees from Molecular Partners; honoraria from Medicine Journal; funding from MRC, Kidney Research Yorkshire, Kidney Research UK, and Leeds Cares for earlier studies relating to this research; and was previously British Transplant Society Ethics Committee Chair and NHS Blood and Transplant Environmental Sustainability Working Group Chair. All the other authors declared no competing interests.

## Supplementary Material

Supplementary File (PDF) Figure S1. Kaplan Meier curves for the discovery and validation phase cohorts showing death-censored graft survival (DCGS) for the patients based on the clinical and demographic variables available at the time of transplant and additionally for 1 year eGFR for comparison. Figure S2. Correlation of assay results for ACY1 (*n* = 377) between the original Leeds ELISA and the prototype Randox biochip (Spearman’s rank correlation coefficient = 0.95, p<2.2 x e-16) with (A) the full range of results, and (B) an expanded x axis to allow the lower values to be seen more clearly. Figure S3. Distribution plots of the biomarkers measured in days 1 to 3 posttransplant serum samples in phase 1. Figure S4. Biomarker-stratified Kaplan Meier curves for the deceased donor kidney transplant (DDKT) patients (DGF and non-DGF subgroups) in the discovery and the validation cohorts. Table S1. Discovery and validation phase cohorts showing the numbers of patients from each cohort included in the DGF prediction or prognostic analyses based on sample availability for the relevant days posttransplant (day 0 is the day of transplant based on reperfusion of the kidney). Table S2. Randox biochip assay details as used in the discovery phase. Table S3. Associations in the discovery phase between clinical and demographic variables and biomarker concentrations measured in serum samples at days 1 to 3 posttransplant and considered for inclusion in the linear predictor modelling. Table S4. Initial variable selection for the linear predictor for prediction of DGF and DCGS in all transplants using the LASSO model for variable selection. Table S5. Univariable logistic regression examining associations of clinical parameters and serum biomarkers (day 1 or 2 posttransplant) with DGF in the discovery phase. Table S6. Univariable logistic regression examining associations of clinical parameters and serum biomarkers (day 1, 2, or 3 posttransplant) with DCGS in the discovery phase. Results are shown for (A) all transplants, and (B) deceased donor transplants only. Table S7. Assay details for the RTP biochip developed for the validation phase.
