# Supplementary material for: Biomarker Prediction of Delayed Graft Function and Prognosis Post–Kidney Transplantation
Source: Kidney Int Rep. 2025 Nov 5;11(1):164–86. doi: 10.1016/j.ekir.2025.10.021 (PMC12799578; doi:10.1016/j.ekir.2025.10.021)
Supplement: Supplementary File (PDF) — Figure S1. Kaplan Meier curves for the discovery and validation phase cohorts showing death-censored graft survival (DCGS) for the patients based on the clinical and demographic variables available at the time of transplant and additionally for 1 year eGFR for comparison. Figure S2. Correlation of assay results for ACY1 (n = 377) between the original Leeds ELISA and the prototype Randox biochip (Spearman’s rank correlation coefficient = 0.95, p<2.2 x e-16) with (A) the full range of results, and (B) an expanded x axis to allow the lower values to be seen more clearly. Figure S3. Distribution plots of the biomarkers measured in days 1 to 3 posttransplant serum samples in phase 1. Figure S4. Biomarker-stratified Kaplan Meier curves for the deceased donor kidney transplant (DDKT) patients (DGF and non-DGF subgroups) in the discovery and the validation cohorts. Table S1. Discovery and validation phase cohorts showing the numbers of patients from each cohort included in the DGF prediction or prognostic analyses based on sample availability for the relevant days posttransplant (day 0 is the day of transplant based on reperfusion of the kidney). Table S2. Randox biochip assay details as used in the discovery phase. Table S3. Associations in the discovery phase between clinical and demographic variables and biomarker concentrations measured in serum samples at days 1 to 3 posttransplant and considered for inclusion in the linear predictor modelling. Table S4. Initial variable selection for the linear predictor for prediction of DGF and DCGS in all transplants using the LASSO model for variable selection. Table S5. Univariable logistic regression examining associations of clinical parameters and serum biomarkers (day 1 or 2 posttransplant) with DGF in the discovery phase. Table S6. Univariable logistic regression examining associations of clinical parameters and serum biomarkers (day 1, 2, or 3 posttransplant) with DCGS in the discovery phase. Results are shown for (A) all t [file mmc1.pdf]

TRIPOD Checklist: Prediction Model Development and Validation

Section/Topic	Item		Checklist Item	Page
**Title and abstract**				
Title	1	D;V	Identify the study as developing and/or validating a multivariable prediction model, the target population, and the outcome to be predicted.	1
Abstract	2	D;V	Provide a summary of objectives, study design, setting, participants, sample size, predictors, outcome, statistical analysis, results, and conclusions.	4
**Introduction**				
Background and objectives	3a	D;V	Explain the medical context (including whether diagnostic or prognostic) and rationale for developing or validating the multivariable prediction model, including references to existing models.	5
	3b	D;V	Specify the objectives, including whether the study describes the development or validation of the model or both.	5
**Methods**				
Source of data	4a	D;V	Describe the study design or source of data (e.g., randomized trial, cohort, or registry data), separately for the development and validation data sets, if applicable.	6
	4b	D;V	Specify the key study dates, including start of accrual; end of accrual; and, if applicable, end of follow-up.	6
Participants	5a	D;V	Specify key elements of the study setting (e.g., primary care, secondary care, general population) including number and location of centres.	6
	5b	D;V	Describe eligibility criteria for participants.	6
	5c	D;V	Give details of treatments received, if relevant.	6
Outcome	6a	D;V	Clearly define the outcome that is predicted by the prediction model, including how and when assessed.	7
	6b	D;V	Report any actions to blind assessment of the outcome to be predicted.	6
Predictors	7a	D;V	Clearly define all predictors used in developing or validating the multivariable prediction model, including how and when they were measured.	8
	7b	D;V	Report any actions to blind assessment of predictors for the outcome and other predictors.	8
Sample size	8	D;V	Explain how the study size was arrived at.	8, 10
Missing data	9	D;V	Describe how missing data were handled (e.g., complete-case analysis, single imputation, multiple imputation) with details of any imputation method.	7
Statistical analysis methods	10a	D	Describe how predictors were handled in the analyses.	8-10
	10b	D	Specify type of model, all model-building procedures (including any predictor selection), and method for internal validation.	8-10
	10c	V	For validation, describe how the predictions were calculated.	8-10
	10d	D;V	Specify all measures used to assess model performance and, if relevant, to compare multiple models.	9
	10e	V	Describe any model updating (e.g., recalibration) arising from the validation, if done.	N/A
Risk groups	11	D;V	Provide details on how risk groups were created, if done.	9-10
Development vs. validation	12	V	For validation, identify any differences from the development data in setting, eligibility criteria, outcome, and predictors.	10
**Results**				
Participants	13a	D;V	Describe the flow of participants through the study, including the number of participants with and without the outcome and, if applicable, a summary of the follow-up time. A diagram may be helpful.	6,10-11,13
	13b	D;V	Describe the characteristics of the participants (basic demographics, clinical features, available predictors), including the number of participants with missing data for predictors and outcome.	10-11, 13, 7-8
	13c	V	For validation, show a comparison with the development data of the distribution of important variables (demographics, predictors and outcome).	13, 14
Model development	14a	D	Specify the number of participants and outcome events in each analysis.	10-12
	14b	D	If done, report the unadjusted association between each candidate predictor and outcome.	–
Model specification	15a	D	Present the full prediction model to allow predictions for individuals (i.e., all regression coefficients, and model intercept or baseline survival at a given time point).	12
	15b	D	Explain how to the use the prediction model.	12
Model performance	16	D;V	Report performance measures (with CIs) for the prediction model.	12-14
Model-updating	17	V	If done, report the results from any model updating (i.e., model specification, model performance).	N/A
**Discussion**				
Limitations	18	D;V	Discuss any limitations of the study (such as nonrepresentative sample, few events per predictor, missing data).	16, 18
Interpretation	19a	V	For validation, discuss the results with reference to performance in the development data, and any other validation data.	15-16
	19b	D;V	Give an overall interpretation of the results, considering objectives, limitations, results from similar studies, and other relevant evidence.	15-18
Implications	20	D;V	Discuss the potential clinical use of the model and implications for future research.	16-18
**Other information**				
Supplementary information	21	D;V	Provide information about the availability of supplementary resources, such as study protocol, Web calculator, and data sets.	19, 28
Funding	22	D;V	Give the source of funding and the role of the funders for the present study.	19, 20

*Items relevant only to the development of a prediction model are denoted by D, items relating solely to a validation of a prediction model are denoted by V, and items relating to both are denoted D;V. We recommend using the TRIPOD Checklist in conjunction with the TRIPOD Explanation and Elaboration document.

SUPPLEMENTARY FIGURES

Supplementary Figure S1. Kaplan Meier curves for the discovery and validation phase cohorts showing death-censored graft survival (DCGS) for the patients based on the clinical and demographic variables available at the time of transplant and additionally for 1 year eGFR for comparison a). recipient sex, b). recipient age at transplant, c). recipient ethnicity, d). transplant donor type, e). CIT, f). WIT and g). HLA mismatch, h). previous transplants, i). induction agent, j). maintenance steroids, k). eGFR at 1 year. P values shown are based on log-rank tests. For each variable, K-M curves are provided for the whole cohort and for just the deceased donor kidney transplants (DDKTs).

a). Recipient sex

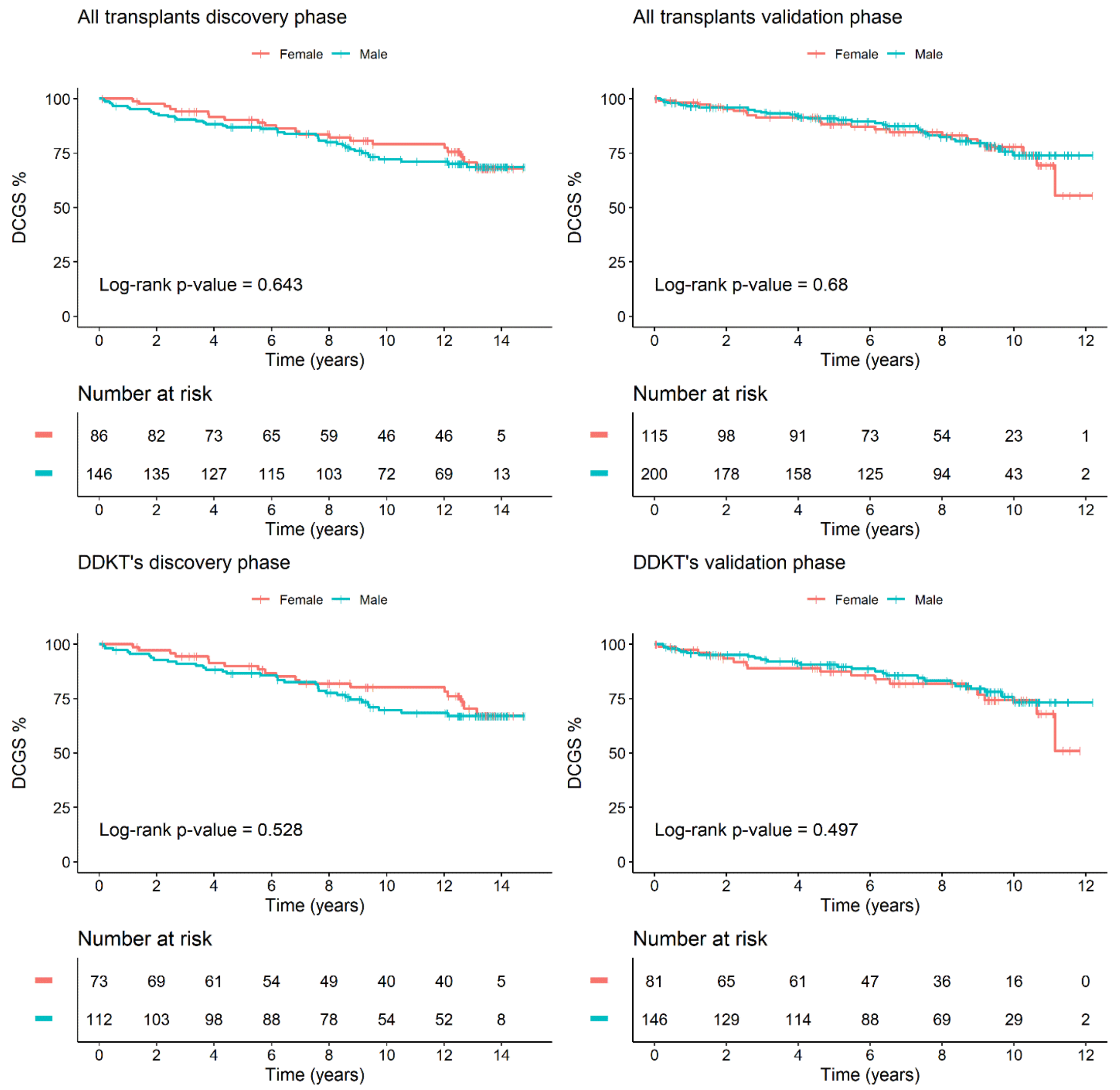

b). Recipient age at transplant (years)

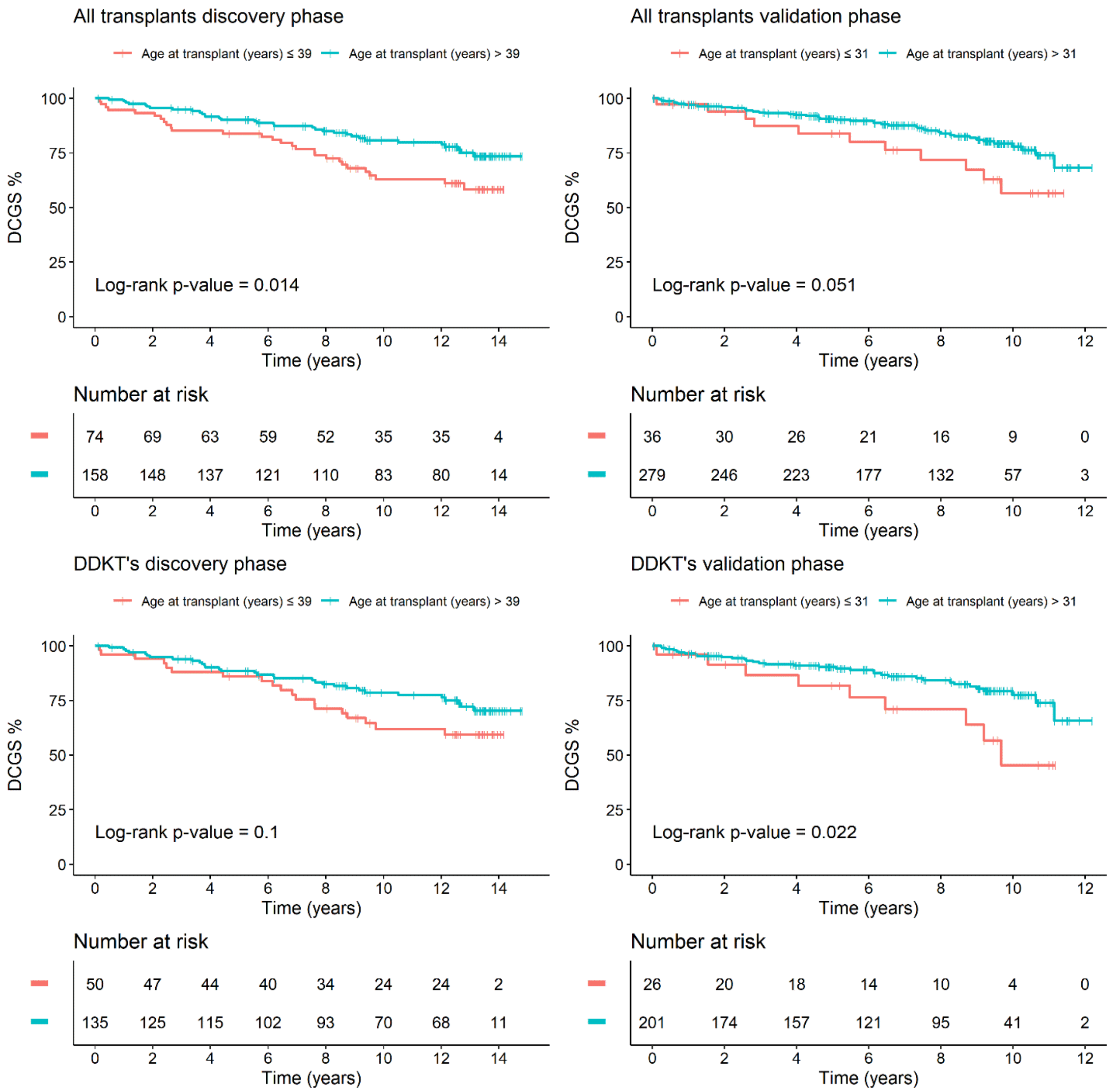

c). Recipient ethnicity

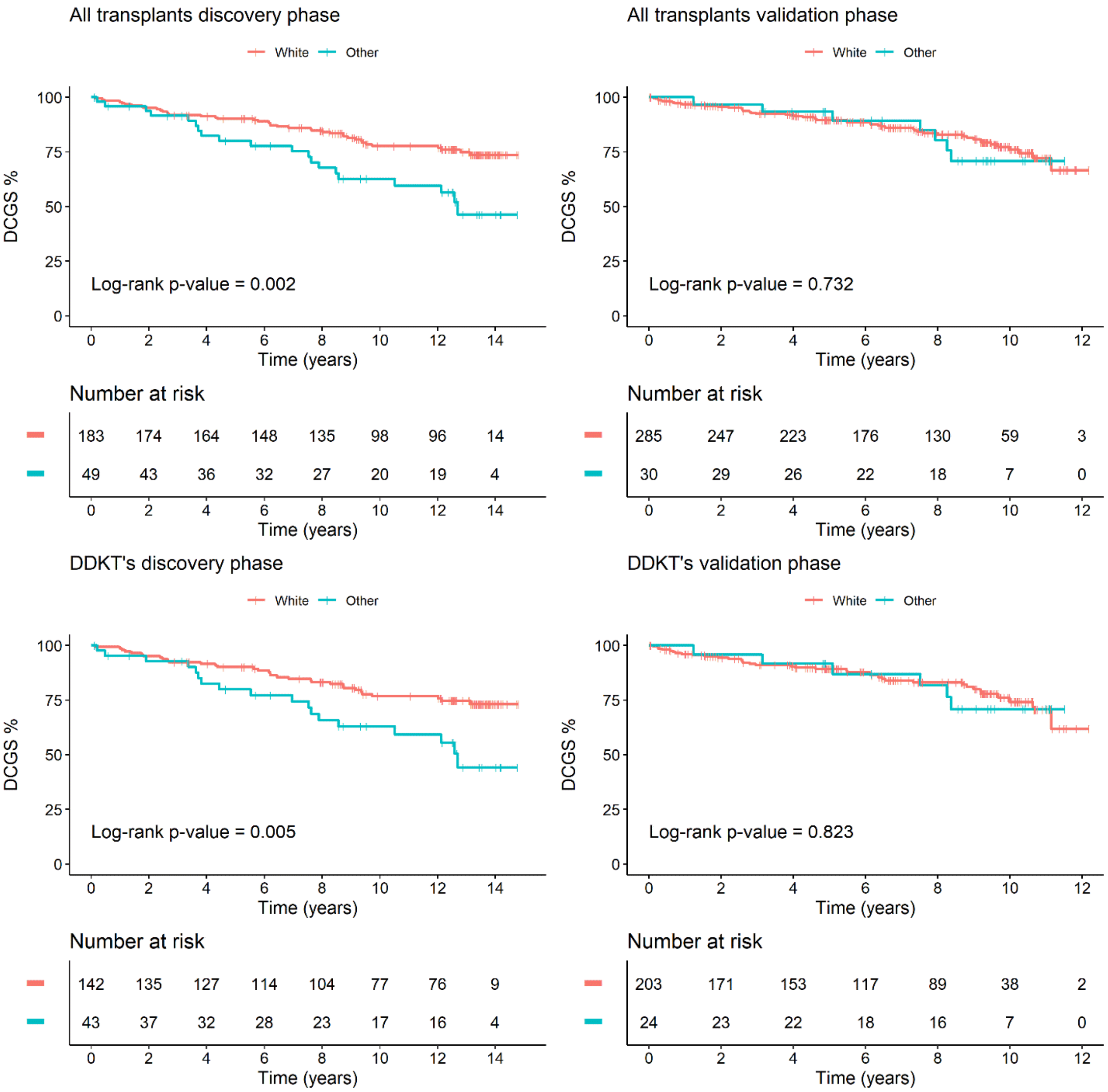

d). Transplant donor type

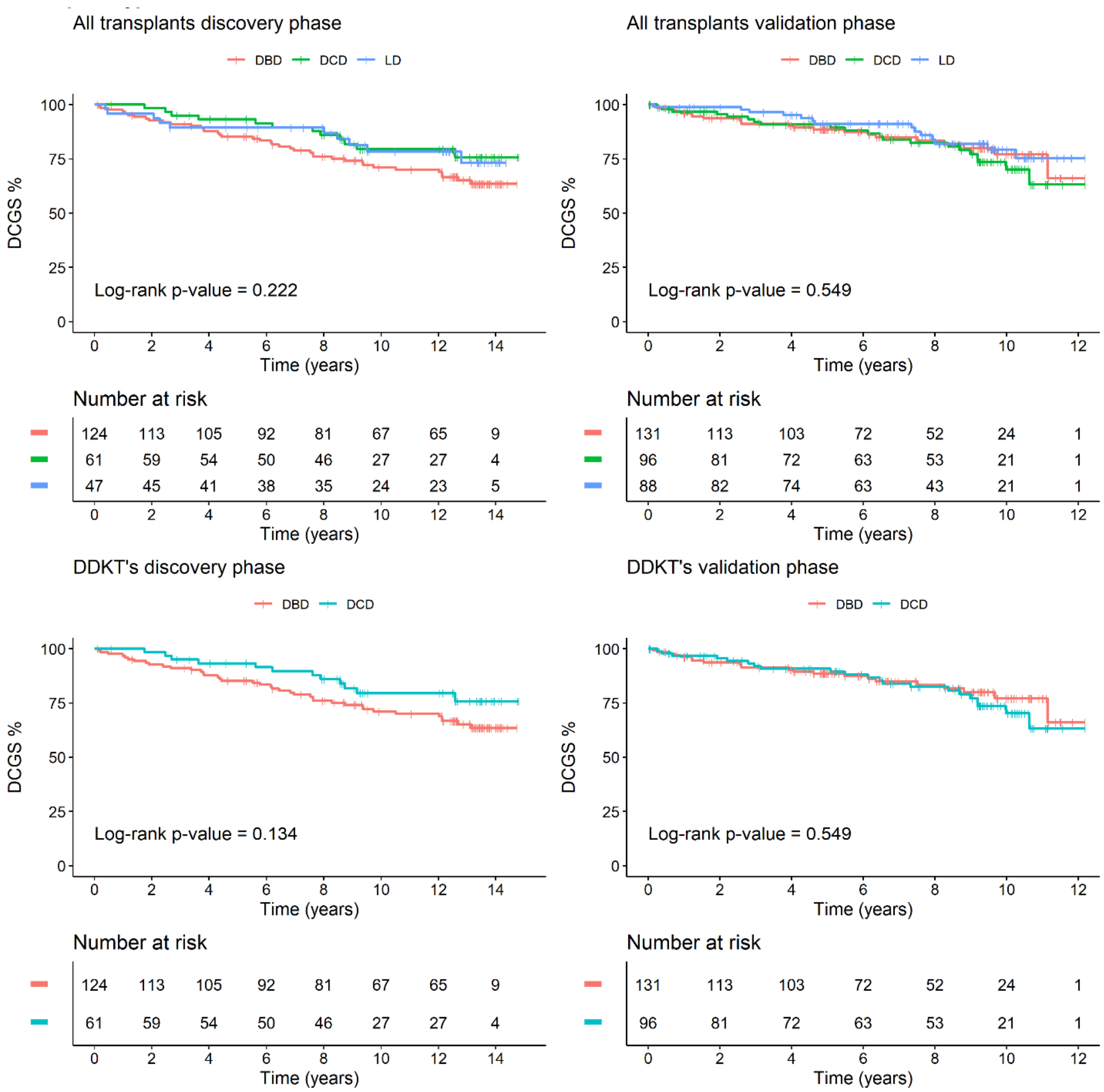

e). CIT

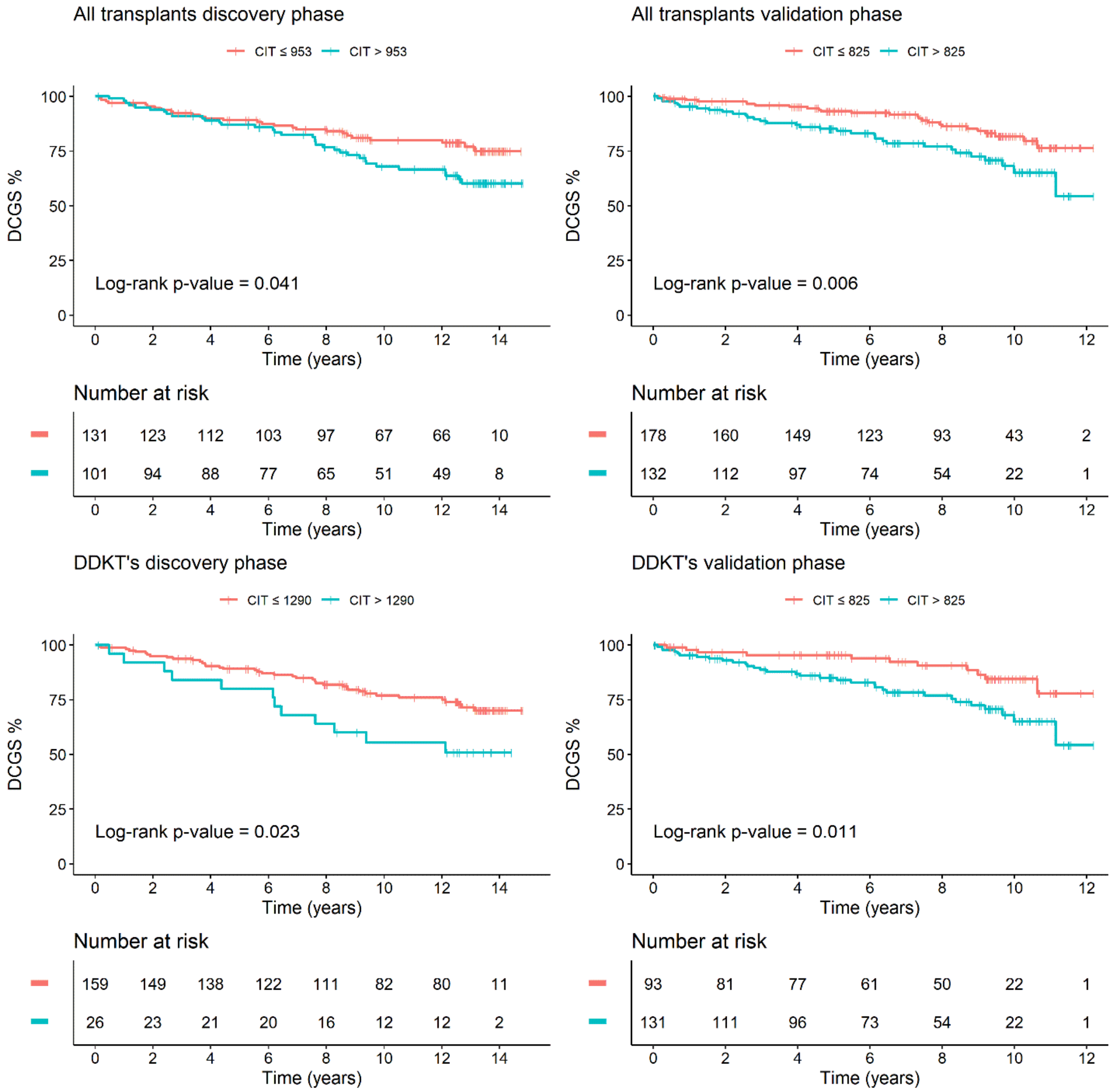

All transplants discovery phase
CIT ≤ 953
CIT > 953
DCGS %
Time (years)
Log-rank p-value = 0.041
Number at risk
131 123 112 103 97 67 66 10
101 94 88 77 65 51 49 8
All transplants validation phase
CIT ≤ 825
CIT > 825
Log-rank p-value = 0.006
178 160 149 123 93 43 2
132 112 97 74 54 22 1
DDKT's discovery phase
CIT ≤ 1290
CIT > 1290
Log-rank p-value = 0.023
159 149 138 122 111 82 80 11
26 23 21 20 16 12 12 2
DDKT's validation phase
CIT ≤ 825
CIT > 825
Log-rank p-value = 0.011
93 81 77 61 50 22 1
131 111 96 73 54 22 1

f). WIT

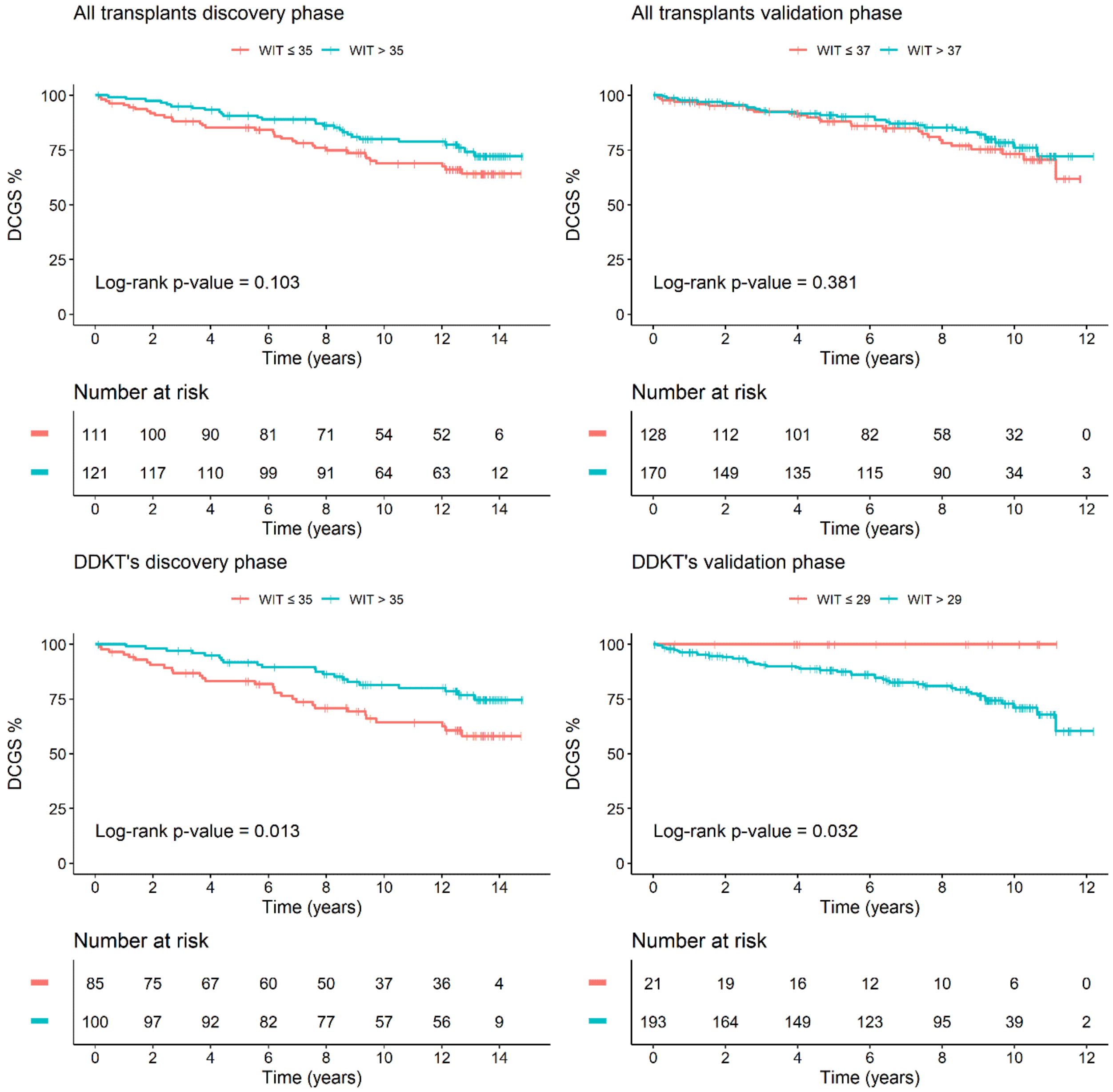
All transplants discovery phase
WIT ≤ 35
WIT > 35
DCGS %
Log-rank p-value = 0.103
Time (years)
Number at risk
111 100 90 81 71 54 52 6
121 117 110 99 91 64 63 12
All transplants validation phase
WIT ≤ 37
WIT > 37
Log-rank p-value = 0.381
Number at risk
128 112 101 82 58 32 0
170 149 135 115 90 34 3
DDKT's discovery phase
WIT ≤ 35
WIT > 35
Log-rank p-value = 0.013
Number at risk
85 75 67 60 50 37 36 4
100 97 92 82 77 57 56 9
DDKT's validation phase
WIT ≤ 29
WIT > 29
Log-rank p-value = 0.032
Number at risk
21 19 16 12 10 6 0
193 164 149 123 95 39 2

g). Total HLA mismatch

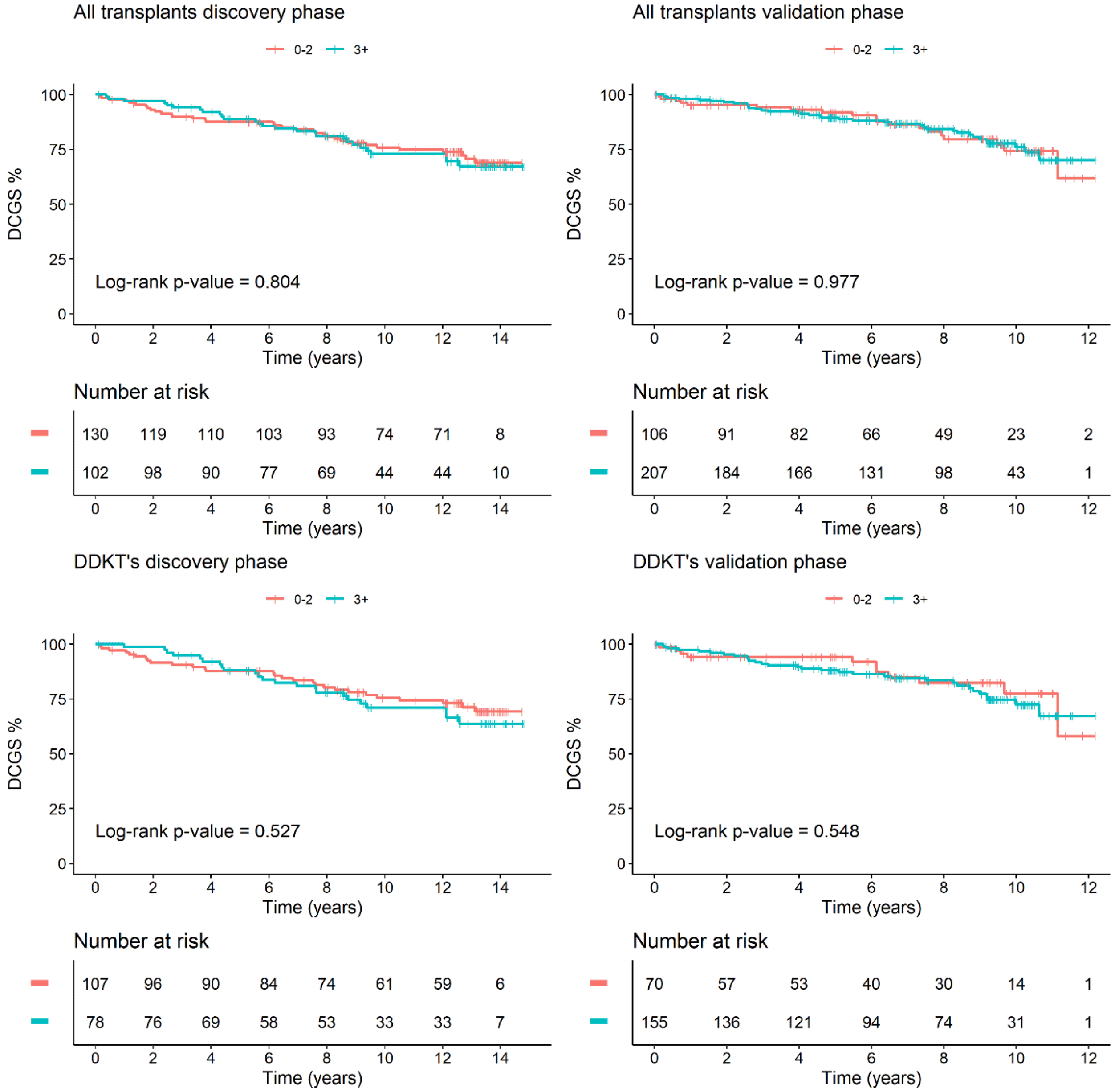

h). Previous transplants

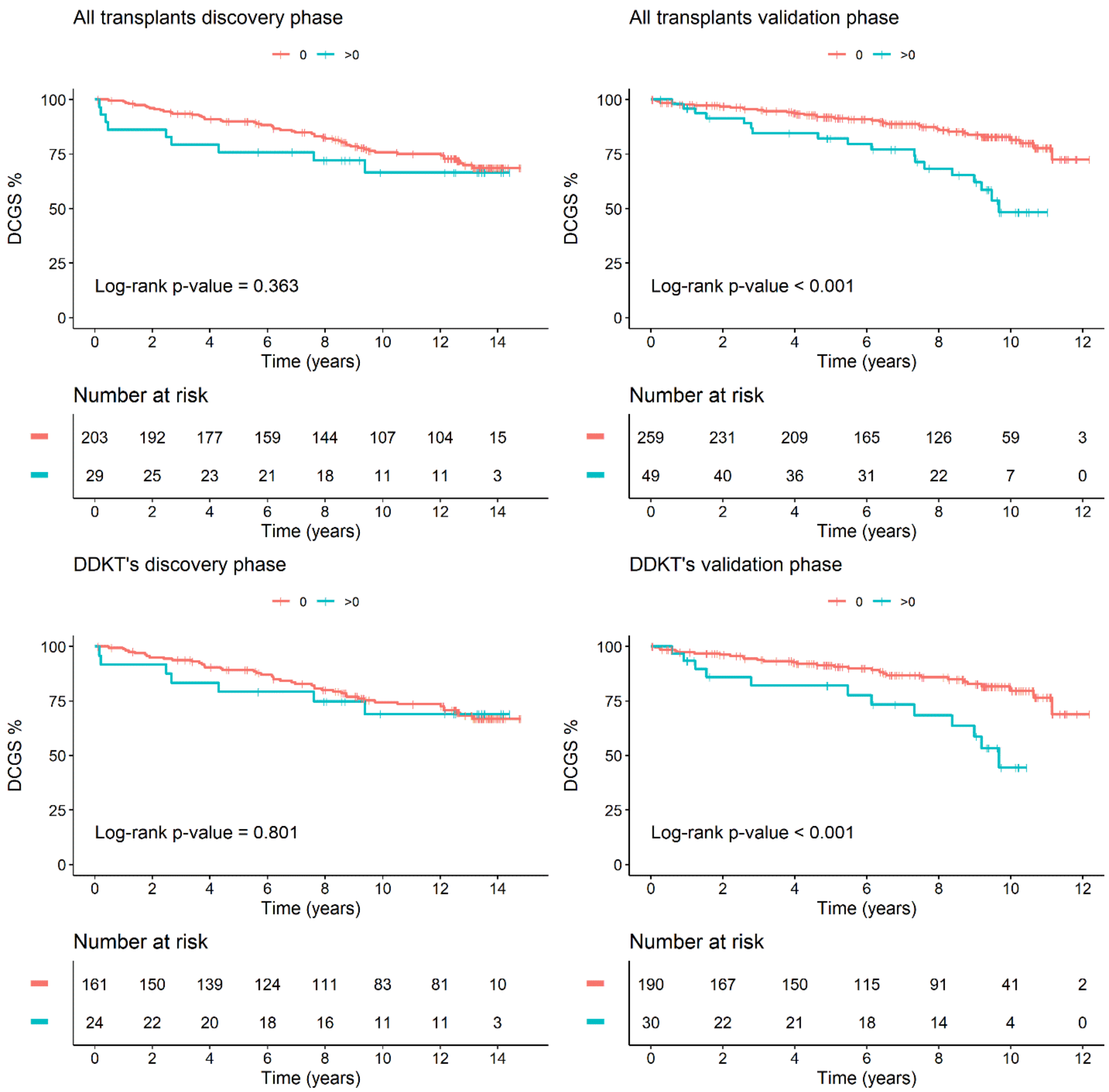

i). Induction agent

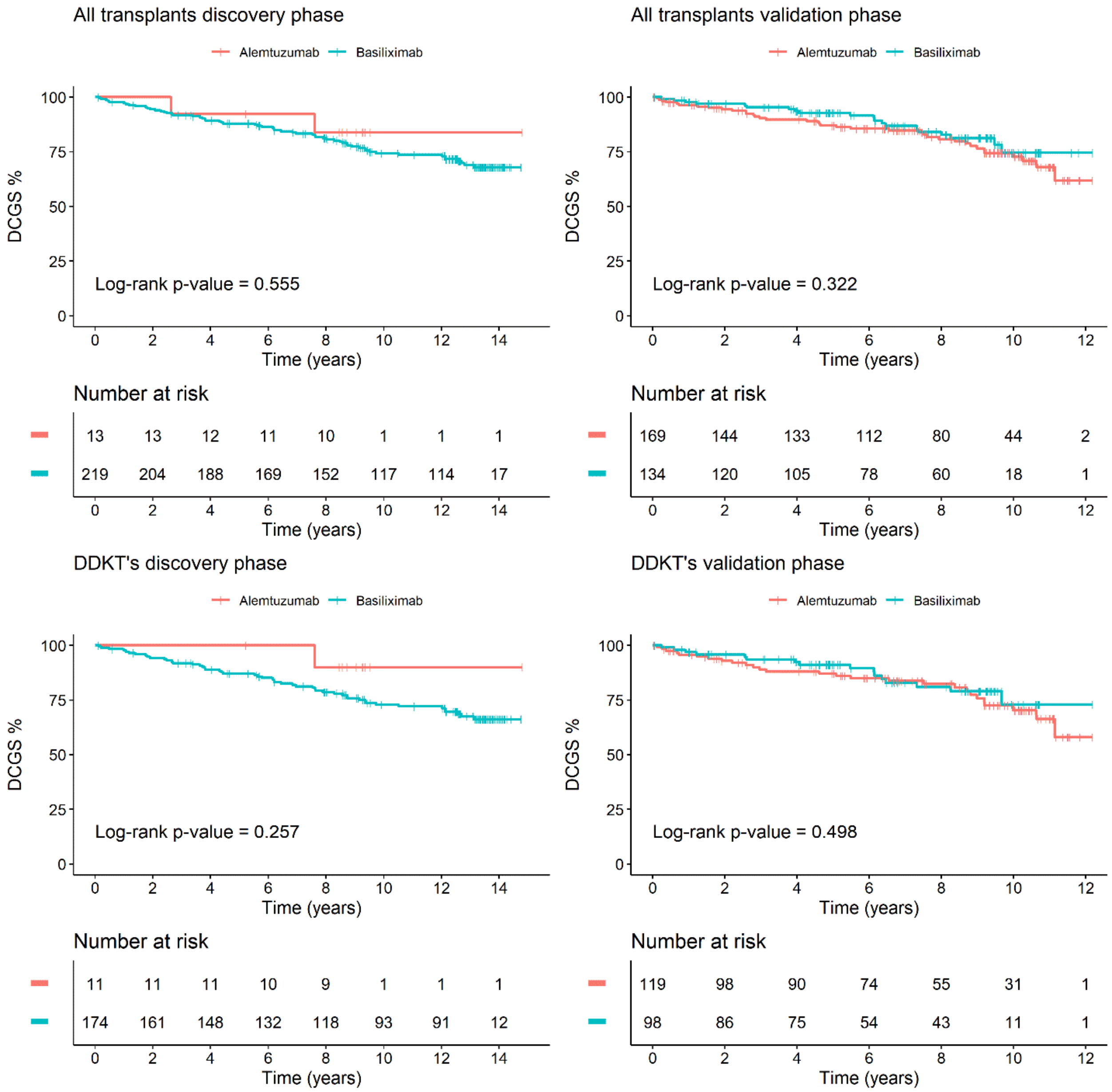

j). Maintenance steroids

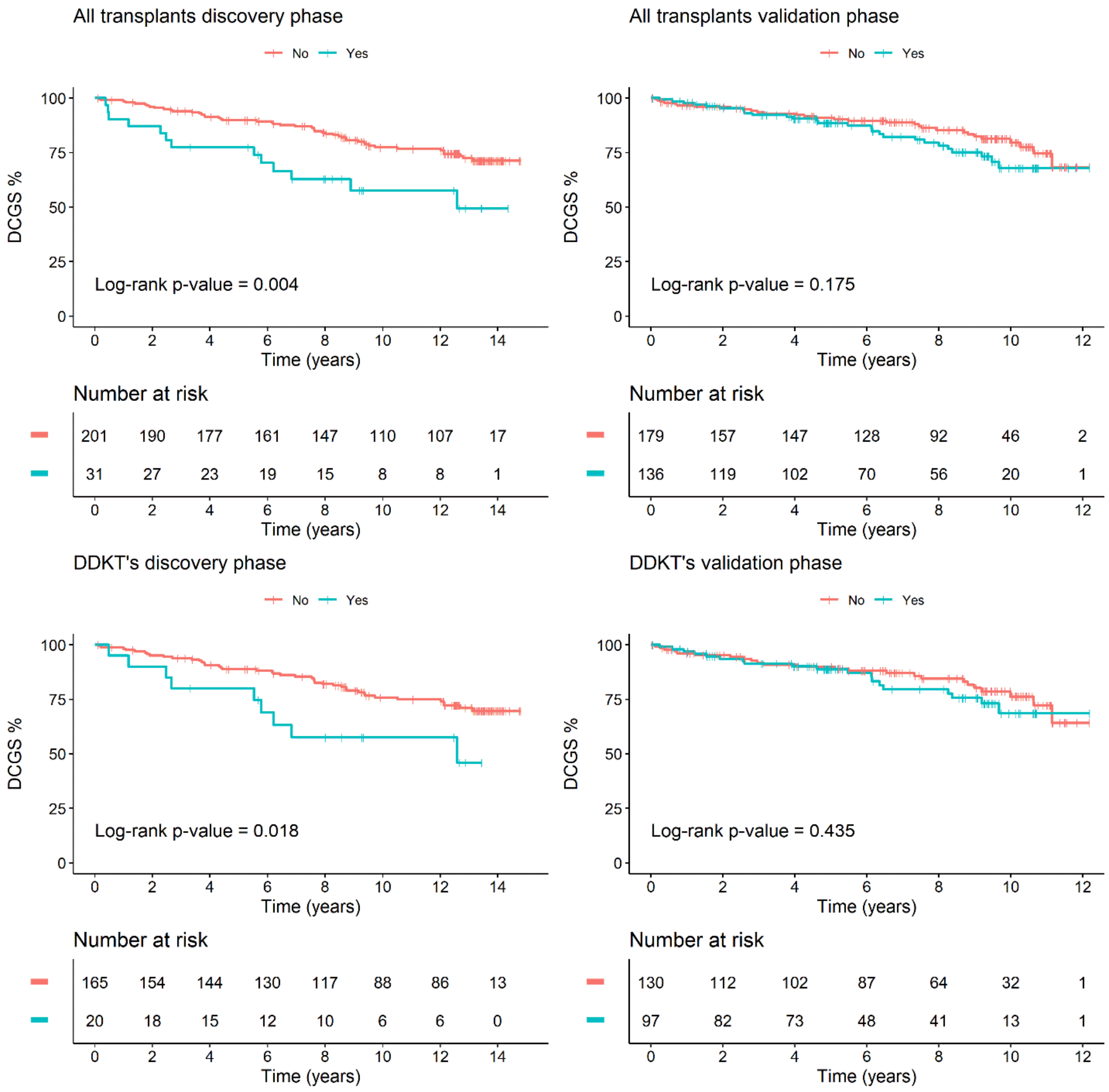

k). eGFR at 1 year

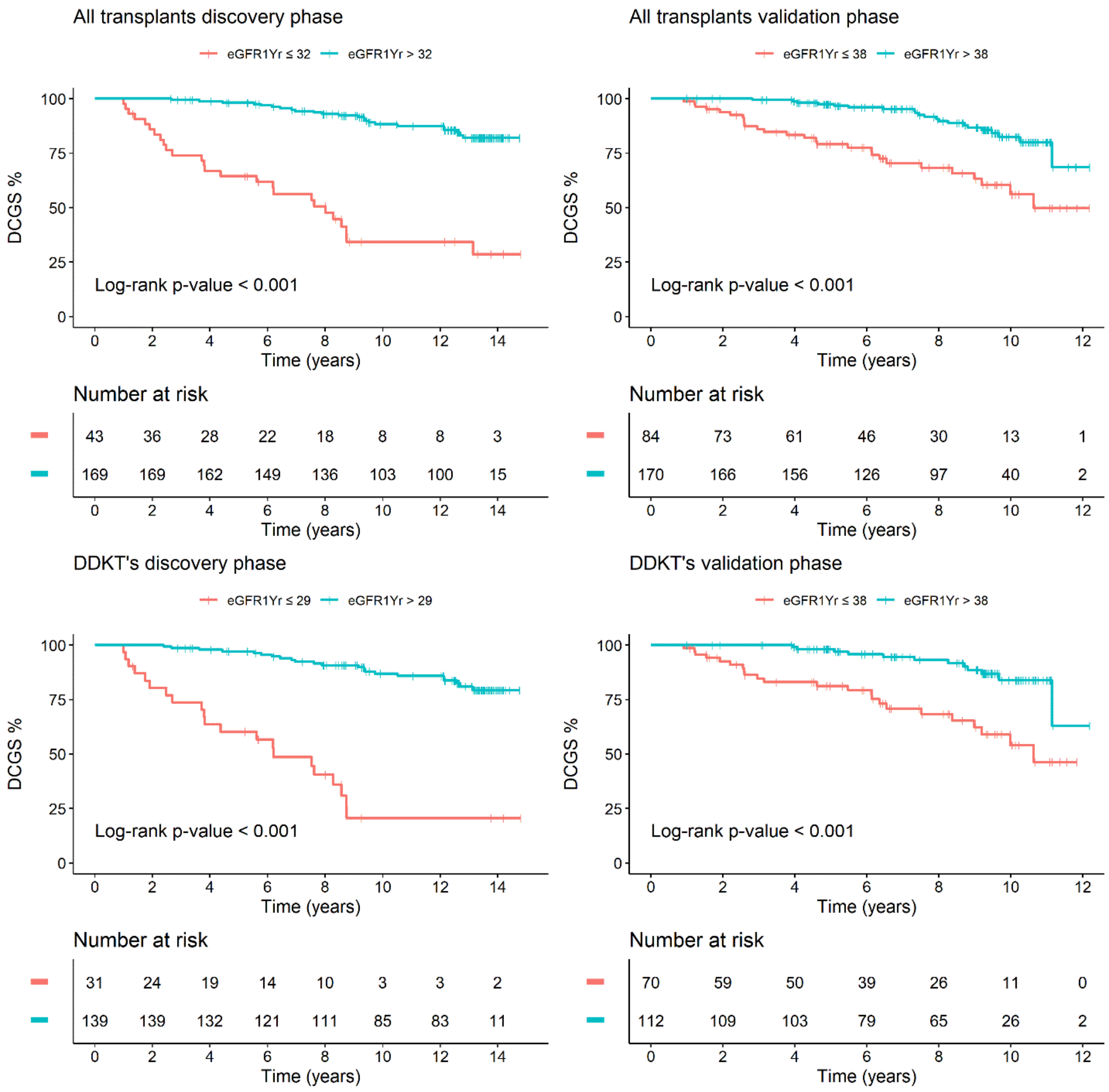

Supplementary Figure S2. Correlation of assay results for ACY1 (n=377) between the original Leeds ELISA and the prototype Randox biochip (Spearman's rank correlation coefficient = 0.95, p<2.2 x e^{-16}) with a). the full range of results, and b). an expanded x axis to allow the lower values to be seen more clearly. Results for two samples identified as outliers were removed as upon re-assay by ELISA different results were obtained compared with the original. Due to differences in standardisation of recombinant ACY-1 materials used for calibration of the two assays, the results generated on the Randox biochip are consistently approximately 50% of those reported by ELISA originally.

a).

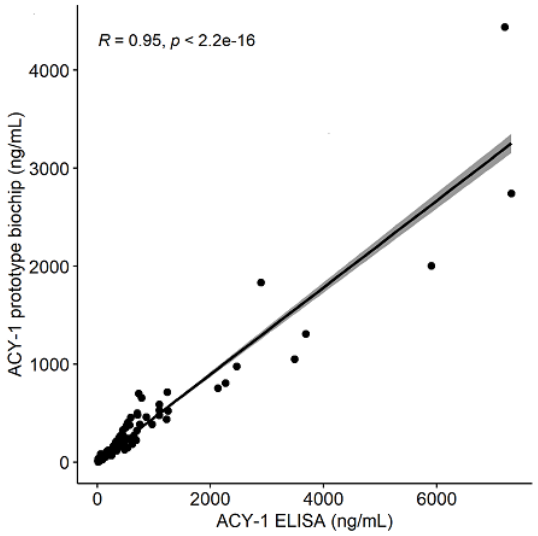

b).

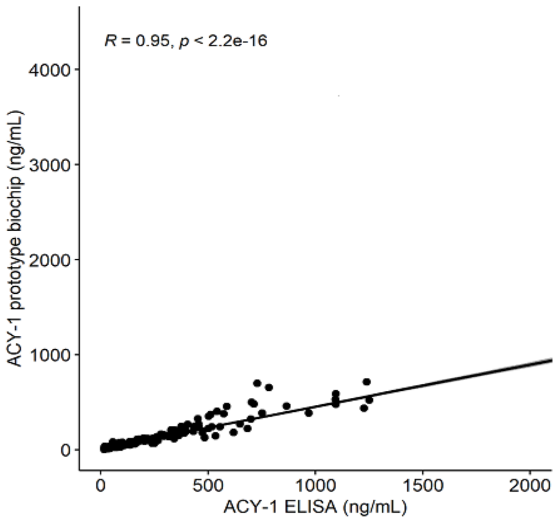

Supplementary Figure S3. Distribution plots of the biomarkers measured in days 1 to 3 post-transplant serum samples in phase 1. Median, upper quartile and lower quartile are shown in black and outliers, defined as values above the upper quartile plus 3 times the interquartile range (IQR) and values below the lower quartile minus 3 times the IQR. Results below the LLOQ were assigned the LLOQ value and of particular note, this involved 37% of MIP-1 alpha results.

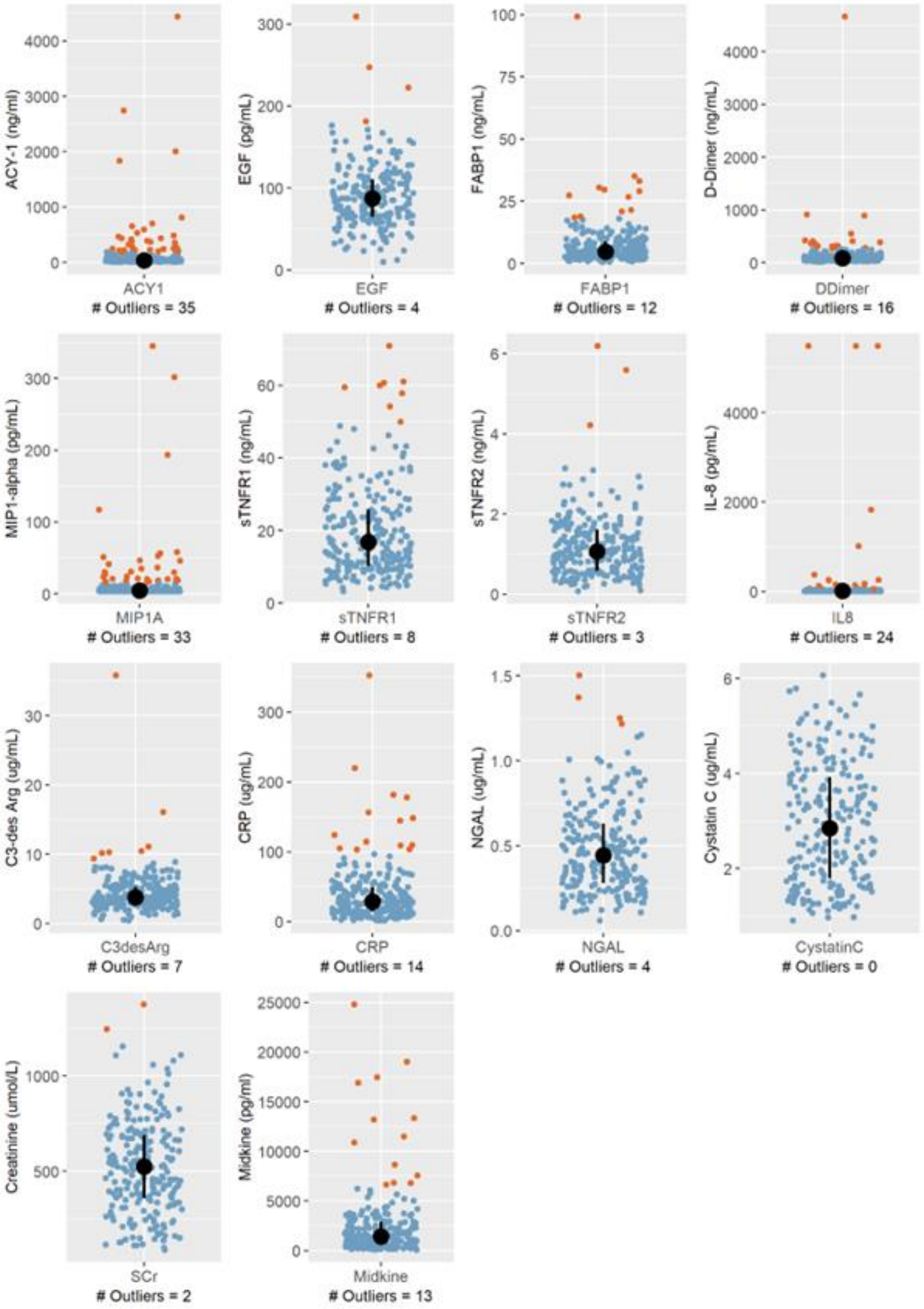

Supplementary Figure S4. Biomarker-stratified Kaplan Meier curves for the deceased donor kidney transplant (DDKT) patients (DGF and non-DGF subgroups) in the discovery cohort and the validation cohorts. The plots show death-censored graft survival (DCGS) with patients stratified by each biomarker initially selected for inclusion in the linear predictor in the discovery phase (sTNFR1, CysC and midkine, with ACY-1 results being shown as Figure 10) with results shown for Cr as the gold standard. *P* values shown are based on log-rank tests.

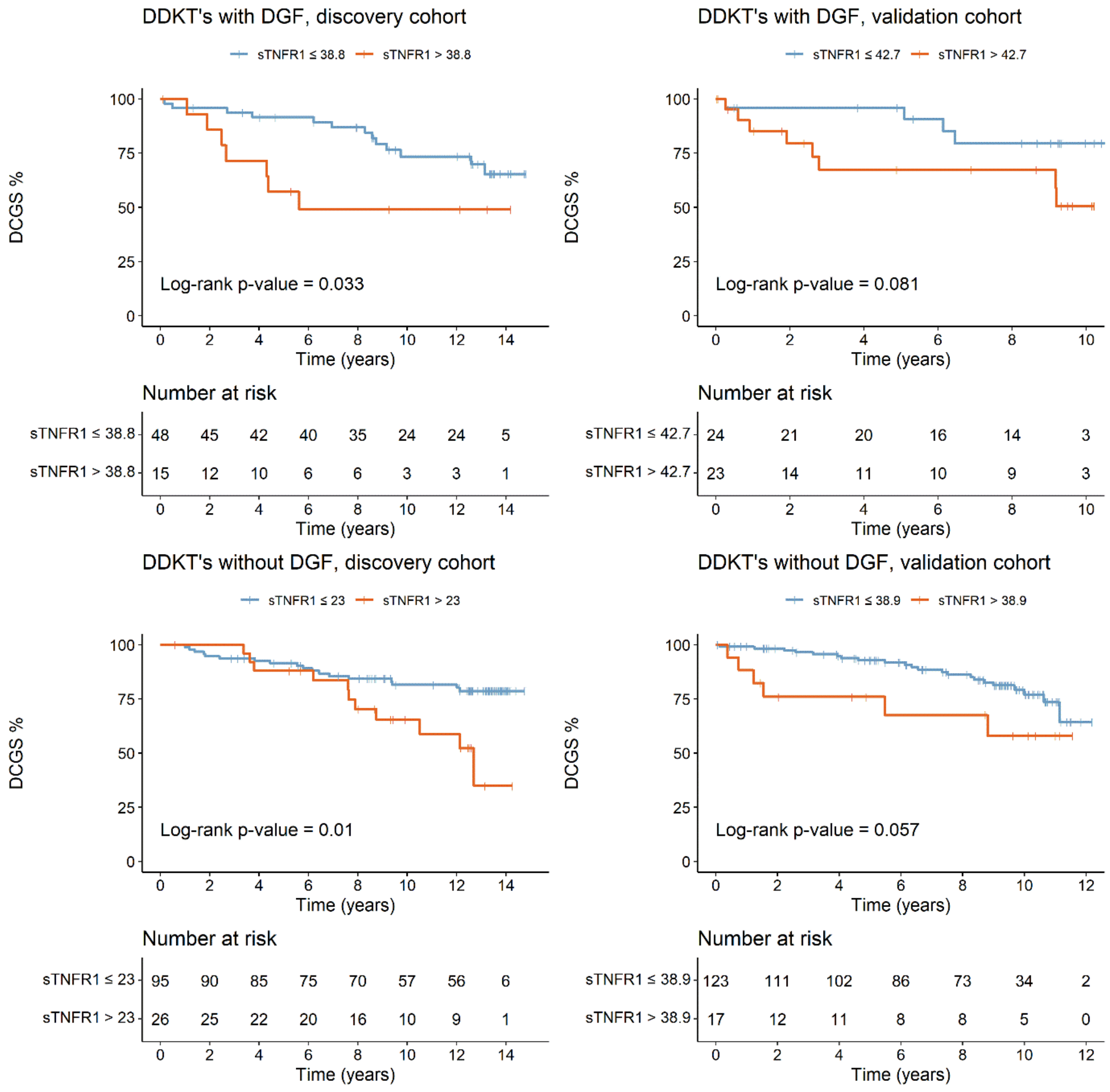

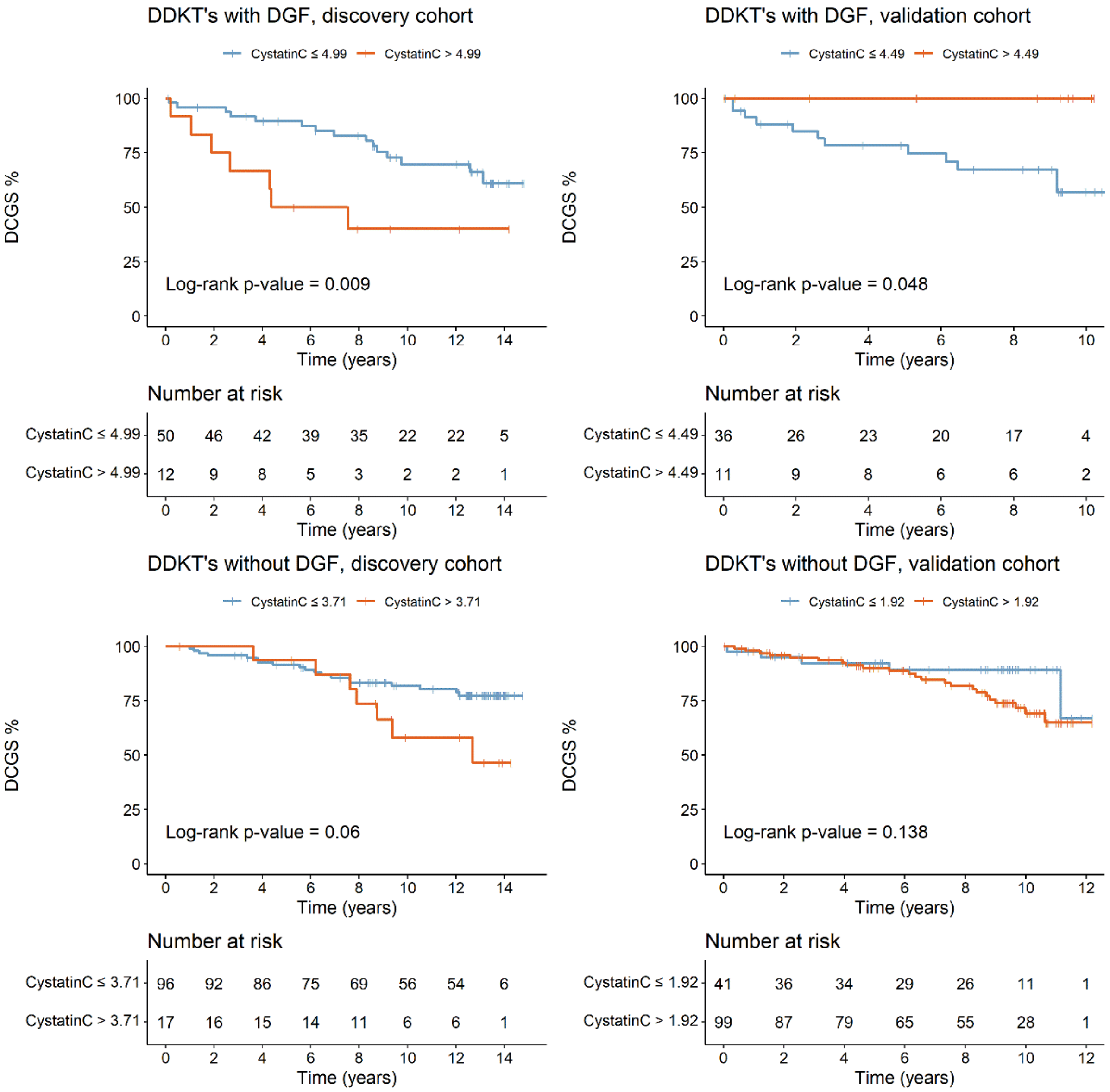
DDKT's with DGF, discovery cohort
CystatinC ≤ 4.99
CystatinC > 4.99
DCGS %
Log-rank p-value = 0.009
Time (years)
Number at risk
CystatinC ≤ 4.99 50 46 42 39 35 22 22 5
CystatinC > 4.99 12 9 8 5 3 2 2 1
DDKT's with DGF, validation cohort
CystatinC ≤ 4.49
CystatinC > 4.49
Log-rank p-value = 0.048
Number at risk
CystatinC ≤ 4.49 36 26 23 20 17 4
CystatinC > 4.49 11 9 8 6 6 2
DDKT's without DGF, discovery cohort
CystatinC ≤ 3.71
CystatinC > 3.71
Log-rank p-value = 0.06
Number at risk
CystatinC ≤ 3.71 96 92 86 75 69 56 54 6
CystatinC > 3.71 17 16 15 14 11 6 6 1
DDKT's without DGF, validation cohort
CystatinC ≤ 1.92
CystatinC > 1.92
Log-rank p-value = 0.138
Number at risk
CystatinC ≤ 1.92 41 36 34 29 26 11 1
CystatinC > 1.92 99 87 79 65 55 28 1

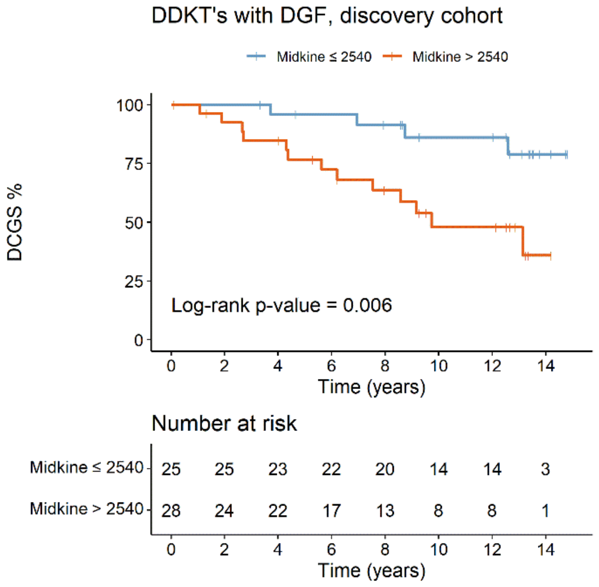
DDKT's with DGF, discovery cohort
Midkine ≤ 2540
Midkine > 2540
DCGS %
100
75
50
25
0
Log-rank p-value = 0.006
0 2 4 6 8 10 12 14
Time (years)
Number at risk
Midkine ≤ 2540 25 25 23 22 20 14 14 3
Midkine > 2540 28 24 22 17 13 8 8 1
0 2 4 6 8 10 12 14
Time (years)

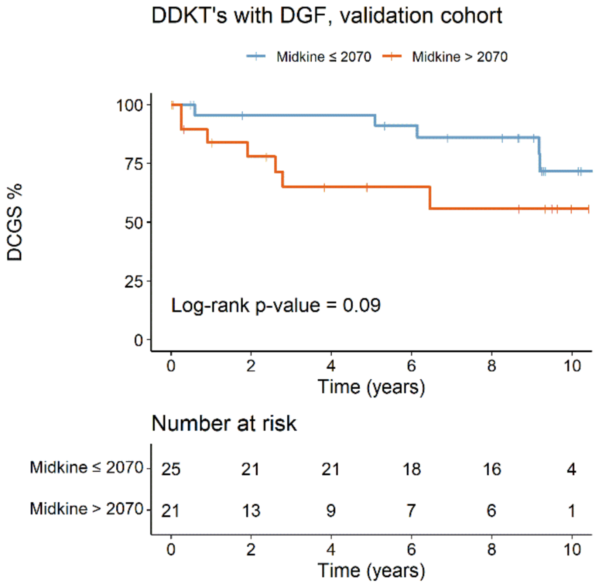
DDKT's with DGF, validation cohort
Midkine ≤ 2070
Midkine > 2070
DCGS %
100
75
50
25
0
Log-rank p-value = 0.09
0 2 4 6 8 10
Time (years)
Number at risk
Midkine ≤ 2070 25 21 21 18 16 4
Midkine > 2070 21 13 9 7 6 1
0 2 4 6 8 10
Time (years)

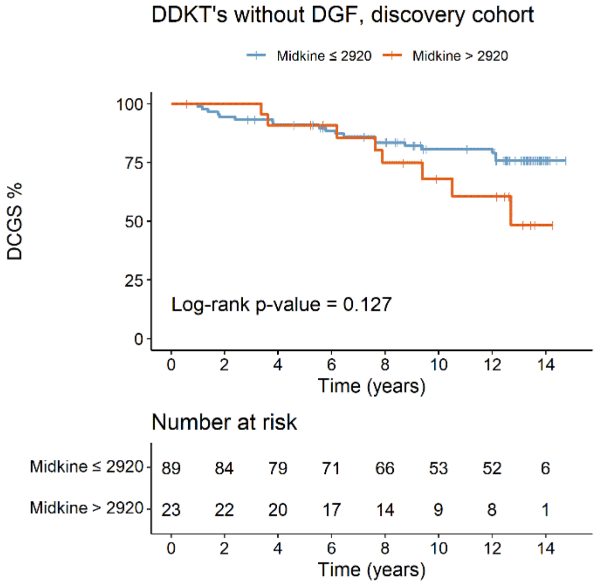
DDKT's without DGF, discovery cohort
Midkine ≤ 2920
Midkine > 2920
DCGS %
100
75
50
25
0
Log-rank p-value = 0.127
0 2 4 6 8 10 12 14
Time (years)
Number at risk
Midkine ≤ 2920 89 84 79 71 66 53 52 6
Midkine > 2920 23 22 20 17 14 9 8 1
0 2 4 6 8 10 12 14
Time (years)

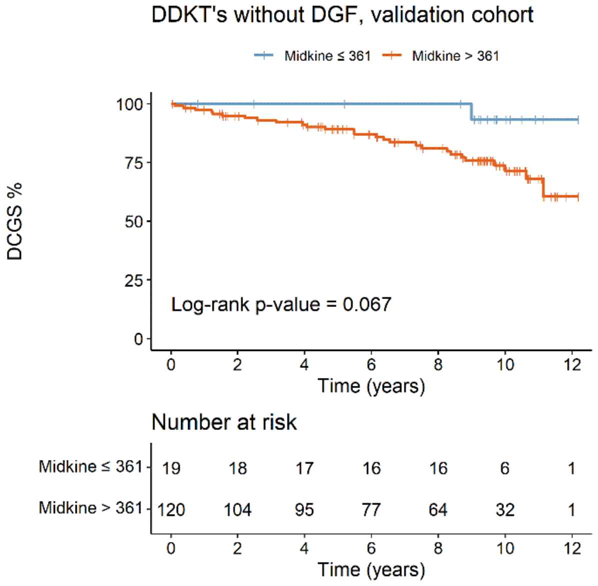
DDKT's without DGF, validation cohort
Midkine ≤ 361
Midkine > 361
DCGS %
100
75
50
25
0
Log-rank p-value = 0.067
0 2 4 6 8 10 12
Time (years)
Number at risk
Midkine ≤ 361 19 18 17 16 16 6 1
Midkine > 361 120 104 95 77 64 32 1
0 2 4 6 8 10 12
Time (years)

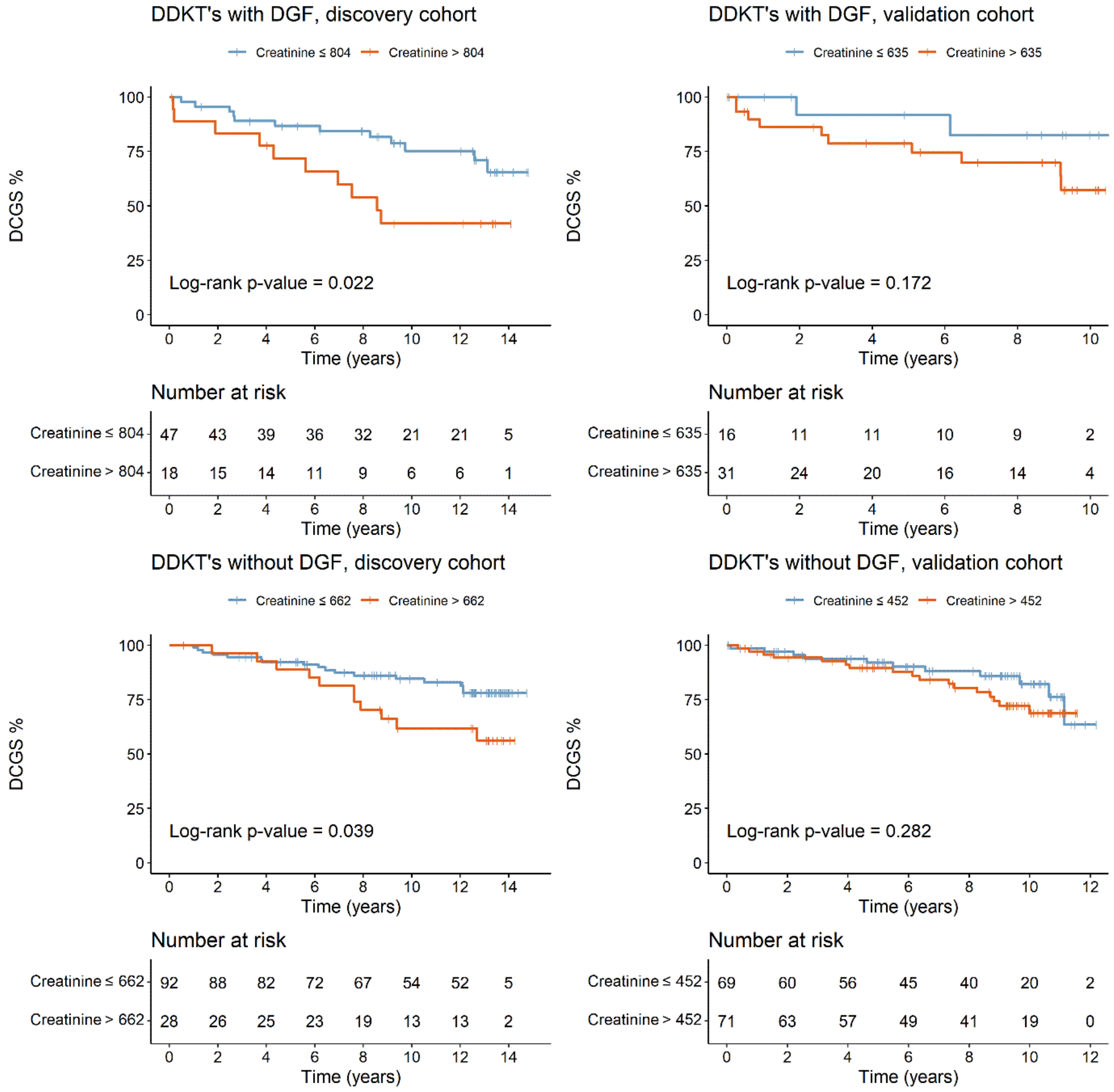
DDKT's with DGF, discovery cohort
Creatinine ≤ 804
Creatinine > 804
DCGS %
Log-rank p-value = 0.022
Time (years)
Number at risk
Creatinine ≤ 804 47 43 39 36 32 21 21 5
Creatinine > 804 18 15 14 11 9 6 6 1
DDKT's with DGF, validation cohort
Creatinine ≤ 635
Creatinine > 635
Log-rank p-value = 0.172
Number at risk
Creatinine ≤ 635 16 11 11 10 9 2
Creatinine > 635 31 24 20 16 14 4
DDKT's without DGF, discovery cohort
Creatinine ≤ 662
Creatinine > 662
Log-rank p-value = 0.039
Number at risk
Creatinine ≤ 662 92 88 82 72 67 54 52 5
Creatinine > 662 28 26 25 23 19 13 13 2
DDKT's without DGF, validation cohort
Creatinine ≤ 452
Creatinine > 452
Log-rank p-value = 0.282
Number at risk
Creatinine ≤ 452 69 60 56 45 40 20 2
Creatinine > 452 71 63 57 49 41 19 0

Supplementary Table S1. Discovery and validation phase cohorts showing the numbers of patients from each cohort included in the DGF prediction or prognostic analyses based on sample availability for the relevant days post-transplant (day 0 is the day of transplant based on reperfusion of the kidney). Additional longitudinal samples up to day 25 were also examined from 22 patients in the discovery phase to examine profiles. (d=day)

Phase 2	Patients in cohort (n)	Prediction of DGF – patients with relevant samples (day 1 or day 2)	Prognosis (DCGS) - patients with relevant samples (days 1, 2 or 3)	Relationship of biomarkers with eGFR - patients with relevant samples (4 weeks +/- 4 days post-transplant)
**Discovery**	237	173* (159 d1 and 14 d2)	237** (159 d1, 14 d2 and 64 d3)	N/A
**Validation**	319	197 (151 d1 and 46 d2)	257 (151 d1, 46 d2 and 60 d3)	276 (includes 62 with no day 1 to 3 samples)

*n=171 with results for all biomarkers included in modelling
**n=232 with results for all biomarkers included in modelling

Supplementary Table S2. Randox biochip assay details as used in the Discovery phase. Range is the range of the standard curve without any dilution after reconstitution of lyophilised standards. Inter-assay precision is also shown calulated from the QC samples across all assay runs in the discovery phase. Dilution is the usual dilution of samples as per the analytical protocol. C3aDesArg and D-dimer assays were also on these biochips but were not used as serum is unsuitable for their measurement.

Biomarker	Abbreviation	Array	Range	Dilution	LLOQ	QC concentrations			%CV			Assay runs (n)
						Control 1	Control 2	Control 3	Control 1	Control 2	Control 3	
**Aminoacylase-1**	ACY-1	ACY-1	0-1000 ng/ml	200x	4.74 ng/ml	17.4	73.3	364.2	11.89	10.63	13.14	39
**Epidermal growth factor**	EGF	CKD1	0-400 pg/ml	neat	6.94 pg/ml	38.4	146.3	261	9.03	9.07	13.24	73
**Fatty acid binding protein 1**	FABP1	CKD1	0-400 ng/ml	neat	0.29 ng/ml	10	47.3	193.1	8.33	8.11	7.8	
**Macrophage inflammatory protein 1-α**	MIP1-α	CKD1	0-1500 pg/ml	neat	3.73 pg/ml	28.2	118	511.6	5.56	4.52	7.61	
**Soluble TNF receptor 1**	sTNFR1	CKD1	0-10 ng/ml	neat	0.04 ng/ml	0.49	1.79	4.65	9.06	6.75	9.27	
**Soluble TNF receptor 2**	sTNFR2	CKD1	0-20 ng/ml	neat	0.07 ng/ml	0.56	1.94	7.84	5.16	5.97	6.43	
**Interleukin-8**	IL-8	CKD1	0-600 pg/ml	neat	3.00 pg/ml	22.4	98.8	427.9	6.87	5.15	12.44	
**C-Reactive protein**	CRP	CKD2	0-60 µg/ml	200x	0.47 µg/ml	1.82	3.53	10.61	5.16	9.01	12.37	54
**Neutrophil gelatinase-associated lipocalin**	NGAL	CKD2	0-2 µg/ml	200x	0.01 µg/ml	0.12	0.45	1.01	10.2	9.69	13.14	
**Cystatin C**	CysC	CKD2	0-5 µg/ml	200x	0.03 µg/ml	0.15	0.47	1.72	8.3	6.4	8.57	

Supplementary Table S3. Associations in the discovery phase between clinical and demographic variables and biomarker concentrations measured in serum samples at days 1 to 3 post-transplant and considered for inclusion in the linear predictor modelling. For the categorical variables data is presented as median (range) and for the continuous variables CIT and WIT the figures shown represent rho.

Characteristic	Level	ACY-1 (ng/ml; n=236)	*P*	EGF (pg/ml; n=223)	*P*	FABP1 (ng/ml; n=225)	*P*	sTNFR1 (ng/mL; n=234)	*P*	IL-8 (pg/mL; n=226)	*P*	NGAL (mg/mL; n=236)	*P*	Cystatin C (ug/ml; n=223)	*P*	Creatinine (umol/L; n=228)	*P*	Midkine (pg/ml; n=214)	*P*
Age at transplant (years)	(-)	0.22	0.001	-0.044	0.510	0.117	0.078	0.162	0.013	0.066	0.327	0.088	0.178	0.19	0.004	-0.023	0.722	0.146	0.033
Sex	Female	36.3 (9.0-4438)	0.342	87.3 (23.7-176)	0.866	5.4 (0.6-99.2)	0.636	16.3 (3.2-61.0)	0.239	15.3 (3.0-1010)	<0.001	0.4 (0.1-1.5)	0.118	2.8 (0.9-5.7)	0.244	421 (98.0-1374)	0.002	1078 (90.0-24785)	0.302
	Male	30.9 (6.5-2741)		86.4 (9.7-309)		4.4 (0.7-35.1)		17.0 (4.2-70.9)		9.9 (3.0-5488)		0.5 (0.1-1.2)		2.8 (1.0-6.1)		552 (87.0-1245)		1484 (75.0-19005)	
Ethnicity	White	33.7 (8.4-4438)	0.934	87.2 (9.7-309)	0.045	4.6 (0.6-99.2)	0.213	16.1 (3.2-70.9)	0.069	11.5 (3.0-5488)	0.285	0.4 (0.1-1.5)	0.031	2.6 (1.0-6.1)	0.005	522 (87.0-1374)	0.004	1326 (90.0-24785)	0.008
	Asian	28.1 (13.1-700)		75.4 (23.3-158)		6.6 (1.3-30.5)		24.7 (4.5-48.8)		16.9 (5.4-1010)		0.6 (0.1-1.2)		3.9 (0.9-5.7)		682 (160-1109)		2437 (564-17459)	
	Black	50.4 (13.7-246)		73.0 (12.1-96.4)		7.3 (1.6-14.1)		17.4 (5.8-32.6)		15.4 (5.6-252)		0.4 (0.2-1.1)		3.1 (1.5-4.2)		604 (149-1245)		973 (320-3957)	
	Other	38.4 (6.5-2005)		103 (33.4-176)		4.6 (1.5-29.6)		13.3 (5.4-37.7)		10.7 (4.0-260)		0.4 (0.1-0.9)		2.4 (1.3-5.0)		407 (143-837)		1335 (75.0-19005)	
Transplant type	DBD	30.9 (8.4-2005)	<0.001	88.7 (9.7-247)	0.313	5.0 (0.6-99.2)	<0.001	18.2 (4.0-70.9)	<0.001	11.7 (3.0-5488)	0.012	0.4 (0.1-1.5)	<0.001	3.1 (1.1-6.1)	<0.001	532 (114-1109)	<0.001	1709 (144-24785)	<0.001
	DCD	155 (14.1-4438)		81.0 (12.1-176)		8.2 (1.6-33.1)		24.6 (5.4-61.0)		14.5 (3.0-5488)		0.6 (0.1-1.4)		3.5 (1.5-5.5)		645 (143-1374)		1820 (75.0-16900)	
	LD	17.0 (6.5-67.0)		86.4 (33.4-309)		2.8 (0.6-13.9)		9.4 (3.2-30.8)		8.8 (3.0-5488)		0.3 (0.1-0.8)		1.5 (0.9-4.7)		361 (87.0-928)		615 (90.0-19005)	
Cause of ESKD	Chronic pyelonephritis	29.7 (9.8-807)	0.825	92.5 (23.7-222)	0.596	5.0 (0.6-33.1)	0.028	16.7 (7.1-59.5)	0.925	10.6 (3.0-125)	0.234	0.4 (0.1-1.4)	0.947	2.5 (1.3-5.7)	0.374	519 (117-928)	0.500	1586 (148-17459)	0.224
	Diabetes	39.3 (11.1-2741)		87.7 (23.3-168)		6.4 (1.4-30.5)		20.1 (7.1-31.4)		13.1 (4.8-57.8)		0.4 (0.1-1.2)		3.6 (1.2-5.7)		464 (129-1109)		2493 (75.0-11488)	
	Glomerulonephritis	37.0 (8.4-4438)		87.2 (9.7-171)		4.7 (0.9-29.1)		16.9 (3.2-70.9)		10.8 (3.0-260)		0.5 (0.1-1.1)		2.6 (1.0-6.1)		526 (110-1374)		1354 (144-24785)	
	Hypertension	24.6 (13.5-246)		75.1 (12.1-309)		2.8 (0.7-13.1)		13.1 (5.8-51.7)		9.1 (5.4-5488)		0.4 (0.2-0.9)		2.4 (1.2-4.5)		413 (126-831)		1064 (239-3461)	
	Inherited	33.3 (6.5-439)		88.7 (33.4-157)		3.3 (0.6-99.2)		14.9 (4.3-61.0)		18.7 (4.0-254)		0.4 (0.1-1.5)		3.2 (1.1-5.5)		545 (98.0-906)		1266 (90.0-19005)	
	Other	43.6 (11.8-152)		78.0 (27.8-158)		5.1 (1.4-12.6)		18.0 (4.2-48.0)		13.6 (3.7-5488)		0.4 (0.2-1.2)		3.5 (0.9-5.3)		573 (148-1106)		1340 (585-13169)	
	Unknown	24.8 (8.6-1833)		92.8 (62.3-247)		5.4 (0.7-18.0)		14.9 (4.9-49.9)		13.4 (3.0-1830)		0.4 (0.1-0.9)		3.2 (1.0-5.8)		573 (87.0-1035)		1806 (113-13345)	
Number of previous transplants	0	33.3 (6.5-4438)	0.592	87.9 (12.1-309)	0.182	4.6 (0.6-99.2)	0.322	15.7 (3.2-70.9)	0.010	12.2 (3.0-5488)	0.641	0.4 (0.1-1.5)	0.602	2.7 (0.9-6.1)	0.022	520 (87.0-1374)	0.115	1335 (75.0-19005)	<0.001
	>0	43.6 (9.8-807)		72.5 (9.7-162)		5.6 (0.6-33.1)		21.0 (7.1-59.5)		11.0 (3.0-371)		0.4 (0.1-1.4)		3.6 (1.3-5.8)		540 (110-1153)		2852 (496-24785)	
Pre-emptive transplant	No	34.8 (8.4-4438)	0.024	86.0 (9.7-247)	0.526	5.0 (0.6-99.2)	<0.001	18.3 (4.0-70.9)	<0.001	12.4 (3.0-5488)	0.181	0.4 (0.1-1.5)	0.122	3.1 (0.9-6.1)	<0.001	543 (87.0-1374)	0.003	1586 (75.0-24785)	<0.001
	Yes	17.5 (6.5-2741)		93.9 (12.1-309)		2.1 (0.6-14.6)		8.1 (3.2-17.7)		8.7 (3.7-5488)		0.4 (0.1-0.8)		1.3 (1.0-2.9)		379 (152-702)		363 (90.0-2490)	
Time on dialysis (mths)	(-)	0.13	0.066	-0.053	0.467	0.209	0.004	0.209	0.003	0.031	0.675	0.133	0.061	0.27	<0.001	0.065	0.361	0.187	0.012
Induction	Alemtuzumab	67.0 (14.1-439)	0.195	67.6 (37.4-114)	0.017	4.0 (1.3-14.1)	0.395	23.6 (5.8-61.0)	0.022	23.0 (5.6-58.5)	0.039	0.7 (0.2-1.1)	0.002	2.5 (1.3-5.2)	0.785	555 (230-906)	0.449	983 (270-6631)	0.360
	Basiliximab	33.3 (6.5-4438)		88.1 (9.7-309)		4.9 (0.6-99.2)		16.5 (3.2-70.9)		11.6 (3.0-5488)		0.4 (0.1-1.5)		2.8 (0.9-6.1)		526 (87.0-1374)		1539 (75.0-24785)	
Maintenance steroids	No	33.1 (6.5-4438)	0.958	87.2 (9.7-309)	0.373	4.8 (0.6-99.2)	0.540	16.9 (4.0-70.9)	0.396	11.5 (3.0-5488)	0.679	0.4 (0.1-1.5)	0.211	2.9 (0.9-6.1)	0.214	528 (87.0-1374)	0.147	1415 (75.0-24785)	0.460
	Yes	40.3 (9.5-590)		87.2 (25.0-171)		3.9 (0.6-33.1)		13.4 (3.2-59.5)		12.6 (3.0-371)		0.4 (0.1-1.4)		1.8 (1.0-5.7)		518 (98.0-1011)		1466 (90.0-6059)	
Total HLA mismatch	0-2	28.1 (6.5-2005)	0.006	87.9 (9.7-247)	0.599	4.6 (0.7-99.2)	0.124	15.2 (3.2-70.9)	0.257	10.7 (3.0-5488)	0.414	0.4 (0.1-1.2)	0.006	2.6 (0.9-6.1)	0.595	516 (110-1374)	0.090	1221 (75.0-8650)	0.071
	3+	40.7 (8.6-4438)		84.9 (12.1-309)		5.0 (0.6-35.1)		18.9 (4.2-61.0)		12.7 (3.0-5488)		0.5 (0.1-1.5)		3.0 (1.0-5.8)		543 (87.0-1245)		1672 (113-24785)	
CIT (hr:mins)	(-)	0.251	<0.001	0.029	0.667	0.23	<0.001	0.33	<0.001	0.142	0.033	0.232	<0.001	0.428	<0.001	0.179	0.006	0.264	<0.001
WIT (mins)	(-)	0.457	<0.001	-0.047	0.480	0.298	<0.001	0.319	<0.001	0.159	0.017	0.235	<0.001	0.229	0.001	0.194	0.003	0.062	0.366
DGF status	No	24.3 (6.5-657)	<0.001	87.3 (12.1-309)	0.193	3.8 (0.6-99.2)	<0.001	12.7 (3.2-61.0)	<0.001	10.4 (3.0-5488)	0.002	0.3 (0.1-1.2)	<0.001	2.1 (0.9-5.5)	<0.001	424 (87.0-1059)	<0.001	898 (75.0-19005)	<0.001
	Yes	128 (9.8-4438)		84.9 (9.7-168)		8.7 (2.9-33.1)		30.5 (14.9-70.9)		15.3 (4.8-1010)		0.7 (0.3-1.5)		4.3 (2.6-6.1)		686 (358-1374)		2820 (719-24785)	

Supplementary Table S4. Initial variable selection for the linear predictor for prediction of DGF and DCGS in all transplants using the LASSO model for variable selection. This results in two models for commonly used tuning parameters (lambda 1SE and lambda min).

Biomarker	lambda = 1SE	lambda = min	lambda = 1SE	lambda = min
**(Intercept)**	-12.85	-6.36		
**ACY1**	0.62	0.39	-0.14	-0.06
**EGF**	0.19		0.41	0.24
**FABP1**	0.02		0.01	
**sTNFR1**	0.66	0.54	0.00	
**IL8**	0.04		-0.05	
**NGAL**	0.01		0.19	0.07
**CysC**	3.72	2.10	0.68	0.42
**Cr**	0.14			
**Midkine**	-0.02		0.02	0.01
**Age At Transplant**	0.00		-0.01	0.00
**TransplantType DCD**			-0.08	0.00
**TransplantType LD**				
**Total HLA Mismatch 3+**	0.04			
**CIT**				
**WIT**	0.39			
**Prev transplants >0**			0.00	

Supplementary Table S5. Univariable logistic regression examining associations of clinical parameters and serum biomarkers (day 1 or 2 post-transplant) with DGF in the discovery phase. Results are shown for a). all transplants, and b). deceased donor transplants only. OR = odds ratio, CI = confidence interval

a). All transplants

Predictor	Level	OR	95% CI	p-value
Recipient age (yr)	(-)	1.04	(1.01,1.06)	<0.001
Recipient sex	Female	1		
	Male	0.96	(0.48,1.92)	0.899
Recipient ethnicity	White	1		
	Asian	2.14	(0.84,5.39)	0.106
	Black	2.57	(0.3,22.04)	0.355
	Other	0.77	(0.17,2.68)	0.704
TransplantType	DBD	1		
	DCD	3	(1.45,6.36)	0.004
	LD	0.14	(0.02,0.51)	0.011
Cause of ESKD	Chronic pyelonephritis	1		
	Diabetes	4.2	(1.09,17.91)	0.042
	Glomerulonephritis	1.76	(0.6,5.9)	0.323
	Hypertension	1.68	(0.35,7.77)	0.502
	Inherited	3.44	(0.95,13.68)	0.066
	Other	0.7	(0.09,3.83)	0.696
	Unknown	1.8	(0.46,7.38)	0.399
Number of previous transplants	0	1		
	>0	1.08	(0.39,2.74)	0.878
Pre-emptive transplant	No	1		
	Yes	0.15	(0.01,0.76)	0.068
Time on dialysis (months)	(-)	1.01	(1,1.02)	0.01
Induction	Alemtuzumab	1		
	Basiliximab	1.18	(0.32,5.55)	0.815
Maintenance steroids	No	1		
	Yes	2.34	(0.91,5.95)	0.073
Total HLA mismatch	0-2	1		
	3+	1.95	(1.01,3.8)	0.048
CIT	(-)	1	(1,1)	<0.001
WIT	(-)	1.05	(1.02,1.07)	<0.001
ACY1	(-)	1.01	(1,1.01)	<0.001
EGF	(-)	1	(0.99,1.01)	0.95
FABP1	(-)	1.14	(1.08,1.22)	<0.001
sTNFR1	(-)	1.15	(1.1,1.21)	<0.001
IL8	(-)	1	(1,1)	0.78
NGAL	(-)	18.95	(7.32,56.96)	<0.001
CystatinC	(-)	6.71	(3.9,13.13)	<0.001
Creatinine	(-)	1	(1,1.01)	<0.001
Midkine	(-)	1	(1,1)	0.02

b). Deceased donor transplants

Predictor	Level	OR	95% CI	p-value
Recipient age (yr)	(-)	1.03	(1.01,1.06)	0.02
Recipient sex	Female	1		
	Male	0.95	(0.46,2)	0.901
Recipient ethnicity	White	1		
	Asian	2.06	(0.76,5.66)	0.151
	Black	1.86	(0.22,16.02)	0.545
	Other	0.8	(0.16,3.06)	0.752
TransplantType	DBD	1		
	DCD	3	(1.45,6.36)	0.004
	LD			
Cause of ESKD	Chronic pyelonephritis	1		
	Diabetes	3.73	(0.87,17.61)	0.082
	Glomerulonephritis	2.06	(0.67,7.18)	0.225
	Hypertension	1.83	(0.36,9.15)	0.456
	Inherited	4.27	(1.03,19.78)	0.051
	Other	0.8	(0.1,4.69)	0.813
	Unknown	1.6	(0.39,6.79)	0.511
Number of previous transplants	0	1		
	>0	0.73	(0.24,2)	0.56
Pre-emptive transplant	No	1		
	Yes	0.26	(0.01,1.6)	0.221
Time on dialysis (months)	(-)	1.01	(1,1.02)	0.12
Induction	Alemtuzumab	1		
	Basiliximab	1.22	(0.31,6)	0.785
Maintenance steroids	No	1		
	Yes	2.44	(0.8,7.87)	0.119
Total HLA mismatch	0-2	1		
	3+	1.93	(0.95,3.96)	0.07
CIT	(-)	1	(1,1)	0.63
WIT	(-)	1.04	(1.01,1.06)	0.01
ACY1	(-)	1.01	(1,1.01)	<0.001
EGF	(-)	1	(0.99,1.01)	0.75
FABP1	(-)	1.1	(1.04,1.17)	<0.001
sTNFR1	(-)	1.13	(1.08,1.19)	<0.001
IL8	(-)	1	(1,1)	0.67
NGAL	(-)	12.32	(4.57,38.7)	<0.001
CystatinC	(-)	5.8	(3.27,11.64)	<0.001
Creatinine	(-)	1	(1,1.01)	<0.001
Midkine	(-)	1	(1,1)	0.06

Supplementary Table S6. Univariable logistic regression examining associations of clinical parameters and serum biomarkers (day 1, 2 or 3 post-transplant) with DCGS in the discovery phase. Results are shown for a). all transplants, and b). deceased donor transplants only. HR = hazard ratio, CI = confidence interval

a). All transplants

Predictor	Level	HR	95% CI	p-value
Recipient age at transplant		0.98	[0.97,1]	0.072
Recipient sex	Female	1		
	Male	1.12	[0.66;1.90]	0.68
Recipient ethnicity	White	1		
	Asian	3.11	[1.69;5.73]	0.001
	Black	0.67	[0.09;4.86]	0.69
	Other	1.69	[0.67;4.28]	0.27
Transplant Type	DBD	1		
	DCD	0.62	[0.32;1.18]	0.14
	LD	0.64	[0.32;1.29]	0.21
ESKD cause	Chronic pyelonephritis	1		
	Diabetes	2.35	[0.81;6.78]	0.12
	Glomerulonephritis	1.95	[0.80;4.75]	0.14
	Hypertension	1.11	[0.31;3.95]	0.87
	Inherited	1.20	[0.39;3.72]	0.75
	Other	1.65	[0.50;5.40]	0.41
	Unknown	1.43	[0.48;4.27]	0.52
Previous transplants	0	1		
	>0	1.39	[0.68;2.81]	0.37
Preemptive	No	1		
	Yes	0.60	[0.19;1.93]	0.39
Time on dialysis (mths)		1	[1,1]	0.00065
Induction	Alemtuzumab	1		
	Basiliximab	1.51	[0.37;6.21]	0.57
Maintenance steroids	No	1		
	Yes	2.44	[1.32;4.52]	0.0046
Total HLA mismatch	0-2	1		
	3+	1.06	[0.64;1.75]	0.83
CIT		1	[1,1]	0.07
WIT		1	[0.98,1]	0.68
ACY1		1	[1,1]	0.22
EGF		1	[1,1]	0.13
FABP1		1	[0.99,1]	0.32
sTNFR1		1	[1,1]	0.05
IL8		1	[0.99,1]	0.47
NGAL		1.8	[1.1,2.9]	0.016
CystatinC		1.4	[1.1,1.7]	0.0016
Creatinine		1	[1,1]	0.015
Midkine		1	[1,1]	0.21

b). Deceased donor transplants

Predictor	Level	HR	95% CI	p-value
Recipient age at transplant		0.99	[0.97,1]	0.29
Recipient sex	Female	1		
	Male	1.20	[0.67;2.13]	0.54
Recipient ethnicity	White	1		
	Asian	2.86	[1.47;5.55]	0.0019
	Black	0.64	[0.09;4.67]	0.66
	Other	2.08	[0.81;5.35]	0.13
Transplant Type	DBD	1		
	DCD	0.62	[0.32;1.18]	0.14
	LD			
ESKD cause	Chronic pyelonephritis	1		
	Diabetes	2.00	[0.67;5.96]	0.22
	Glomerulonephritis	1.33	[0.53;3.35]	0.55
	Hypertension	1.10	[0.31;3.89]	0.88
	Inherited	1.00	[0.31;3.29]	0.99
	Other	1.23	[0.35;4.37]	0.75
	Unknown	1.30	[0.44;3.88]	0.63
Previous transplants	0	1		
	>0	1.11	[0.50;2.46]	0.8
Preemptive	No	1		
	Yes	0.84	[0.20;3.47]	0.81
Time on dialysis (mths)		1	[1,1]	0.036
Induction	Alemtuzumab	1		
	Basiliximab	2.97	[0.41;21.55]	0.28
Maintenance steroids	No	1		
	Yes	2.34	[1.14;4.82]	0.021
Total HLA mismatch	0-2	1		
	3+	1.20	[0.69;2.09]	0.52
CIT		1	[1,1]	0.08
WIT		0.99	[0.97,1]	0.28
ACY1		1	[1,1]	0.17
EGF		1	[1,1]	0.14
FABP1		1	[0.99,1]	0.5
sTNFR1		1	[1,1]	0.13
IL8		1	[0.99,1]	0.49
NGAL		1.9	[1.1,3.2]	0.024
CystatinC		1.4	[1.1,1.9]	0.0059
Creatinine		1	[1,1]	0.039
Midkine		1	[1,1]	0.31

Supplementary Table S7. Assay details for the RTP biochip developed for the validation phase. Range is the range of the standard curve without any dilution. The usual sample dilution was 50x.

Biomarker	Abbreviation	Range	LLOQ
**Aminoacylase-1**	ACY-1	0-500 ng/ml	1.79 ng/ml
**Soluble TNF receptor 1**	sTNFR1	0-80 ng/ml	0.51 ng/ml
**Cystatin C**	CYSC	0-10 µg/ml	0.025 µg/ml
